# Accelerated Screening of Wheat Gluten Strength Using Dual Physicochemical Tests in Diverse Breeding Lines

**DOI:** 10.3390/mps8050124

**Published:** 2025-10-18

**Authors:** Mehri Hadinezhad, Judith Frégeau-Reid, Makayla Giles, Jeremy Ballentine, Brittany Carkner

**Affiliations:** Ottawa Research and Development Centre, Agriculture and Agri-Food Canada, Ottawa, ON K1A 0C6, Canada

**Keywords:** GlutoPeak Tester, protein fractionation, wheat breeding lines, gluten quality assessment, PM-AM

## Abstract

Introducing fast, reliable, and low-input technologies that utilize wholemeal wheat is essential for efficiently screening gluten quality in wheat breeding lines. Although the GlutoPeak Tester (GPT) has been widely studied for gluten assessment, its application in breeding programs remains underexplored. This study presents a comprehensive approach to optimizing a GPT protocol using a diverse set of genotypes collected over seven harvest years and multiple environments. To improve screening capabilities, a quick and simple protein fractionation (PF) technique was integrated into the workflow. Key GPT parameters—such as peak maximum time, maximum torque, and aggregation energy—along with the newly proposed PM-AM parameter, showed strong correlations with established quality traits. PF data, especially insoluble glutenin percentage and the ratio of insoluble to soluble glutenin, provided additional insights into gluten composition. This extensive dataset supports the use of GPT and PF as a dual, high-throughput screening tool. When applied within specific wheat classes and benchmarked against established checks, this method offers a robust strategy for ranking breeding lines based on gluten performance. The use of wholemeal samples further streamlines the process by eliminating the need for milling, making this protocol particularly suitable for early-stage selection in wheat breeding programs.

## 1. Introduction

Wheat remains the cornerstone of global nutrition, with production soaring to over 790 million metric tons in 2024 “https://www.fao.org/worldfoodsituation (accessed on 20 June 2025).” To address the dynamic challenges of climate change and disease, wheat breeding programs focus on developing resilient new genotypes, while farmers seek high-yielding, superior-quality wheat varieties to meet diverse market demands. Early-stage quality screening of breeding lines is a widely accepted strategy, ensuring the advancement of the most promising candidates. When evaluating breeding materials, two main criteria for a successful method are speed and minimal sample size, as numerous samples are likely to be analyzed with limited availability.

Several renowned empirical techniques, such as the Farinograph, Mixograph, Alveograph, and Extensograph, are employed to assess the quality of wheat protein and gluten, which are vital for flour functionalities [1,2,3]. However, these methods necessitate the use of wheat flour, making the milling process indispensable. Additionally, some equipment, like Mixograph, suffers from discontinuity, and the Farinograph data are required to perform the Extensograph, complicating the quality screening of breeding lines. GlutoPeak Tester (GPT) is a relatively new piece of equipment introduced to quickly evaluate the aggregation behavior of wheat gluten under high-speed shearing, providing insights into flour protein functionalities [4,5]. This technique has quickly gained popularity, particularly in breeding programs, due to its speed, low sample requirement, and ability to use wholemeal. Numerous studies have been conducted to assess its merit to evaluate flour functionalities. However, since there is no standardized method approved yet, various approaches have been documented in the literature [6,7,8,9,10,11,12,13,14,15,16,17,18], including a wide range of temperature (20–36 °C), speed (1900–3300 RPM), run time (3–10 min), solvent type (water, without or with 1–5% NaCl salt or CaCl_2_ salt), and different sample/solvent ratios (8.0–11.0 g sample with 1.0–1.4 solvent/sample ratio).

Despite its advantages, the GPT method cannot assess the actual composition of gluten protein. It is well documented that the content and ratio of monomeric and polymeric polypeptides (gliadins and glutenins) as well as insoluble glutenin govern the functionalities observed across different ranges of flour protein strength [3,19,20,21]. A variety of methods have been introduced to fractionate and analyze wheat gluten, including biochemical techniques like SE-HPLC and SDS-PAGE [3,22,23]. These complicated methods are time consuming and expensive and therefore cannot be considered for rapid screening of breeding lines.

To effectively screen wheat breeding lines for gluten profiles, it is essential to develop innovative techniques that are rapid, reliable, require minimal sample sizes, and preferably utilize wholemeal rather than refined flour. This study focused on developing a combined approach using two rapid, complementary physicochemical methods—a wholemeal GPT test and a wholemeal protein fractionation (PF) technique —to characterize the chemical and rheological properties of gluten. We hypothesize that integrating these methods will provide a comprehensive assessment suitable for high-throughput screening in breeding programs. To test this hypothesis, a diverse set of wheat samples, collected over seven harvest years, was analyzed; the results were compared with traditional quality analyses performed on each corresponding sample set to validate the effectiveness of the developed protocols.

## 2. Materials and Methods

### 2.1. Materials

Wheat samples for this study were provided through the wheat registration trials in Ontario, Quebec, and the Maritimes (Canada), as well as wheat breeding materials from the hard winter and hard spring breeding programs at Ottawa Research and Development Centre (ORDC, AAFC, Ottawa, Canada). Various sample sets were tested across multiple locations between 2018 and 2024. Details of each sample set are provided in Section 2.4 and Section 2.5. For each wheat class, “preliminary” breeding lines refer to the F5 or F6 generations derived from parental crosses, while “Advanced” lines correspond to the F7 or F8 generations. These breeding materials were typically bulked from two or three replicates and grown at two or three locations. Registration samples, usually from the F9 or F10 generations, which consisted of bulked materials from six to eight registration trials conducted within each region, with four replications per location. Each trial included one to three check varieties, which are listed in Table 1. All received grains were Dockage cleaned (Dockage Tester, Carter Day International, Minneapolis, MN, USA) before analysis. Chemicals used were all analytical grades from Sigma-Aldrich (MilliporeSigma Canada Ltd., Oakville, ON, Canada).

### 2.2. Wholemeal and Flour Preparation

All wheat registration flour samples were prepared by overnight conditioning the grains to 15.5% moisture content and milling using a Bühler MLU-202 mill (Bühler Holding AG, Uzwil, Switzerland). All wheat breeding samples were milled into flour with a Brabender Quadrumat Junior mill (Anton Paar, Graz, Austria) after overnight conditioning the grains to 15.5% moisture content. The wholemeal samples were prepared using a UDY laboratory mill (UDY Corporation, Fort Collins, CO, USA) with a 0.8 mm sieve without conditioning grains.

### 2.3. Physical Testing

All physical testing was conducted using standard methods approved by the American Association of Cereal Chemists International (AACCI, Approved methods of analysis, 11th ed. AACC Intl., St. Paul, MN, USA; https://www.cerealsgrains.org/resources/methods/Pages/default.aspx (accessed on 15 October 2025)), including Farinograph (Method 54-22.01), Mixograph (Method 54-40.02), Glutomatic (Method 38-12.02), grain and flour protein content (Methods 46-30.01 and 39-10.01), and moisture content (Method 44-15.02). Flour doughs (100 g flour, 14% moisture basis) were prepared and baked according to the Remix method of Canadian Grain Commission methods (https://www.grainscanada.gc.ca/en/grain-research/export-quality/cereals/wheat/methods-tests.html (accessed on 15 October 2025)). Bread volumes (cm^3^) were measured after half-hour cooling at room temperature, using the Volscan machine (Volscan Profiler 300, Stable Micro Systems, Godalming, Surrey, UK).

### 2.4. GlutoPeak Tester (GPT) Protocol

A comprehensive approach was undertaken to develop and optimize the GPT protocol over seven crop harvest years (2018–2024), with the main goal of screening breeding lines. Initially, four different flour samples—a commercial all-purpose flour, two different hard wheat flours from the AACCI Lab Proficiency program check flours, and a soft wheat flour from the AACCI SRC Lab Proficiency Testing check flours—were tested using six different methods (Table 2). Based on the results, the method recommended by Brabender was selected for the second phase, in which samples from the 2018 wheat registration trials in Ontario and Quebec (12 samples from Ontario hard red winter (HRW) wheat, 12 samples from Quebec HRW wheat, 21 samples from Ontario hard spring (HRS) wheat, and 22 samples from Quebec HRS wheat) were analyzed by two labs (Grain Quality Lab, GQL, at Ottawa Research and Development Centre, and the Moulins de Soulanges company in Saint-Polycarpe, QC, Canada) using the same method. In the third stage, a reduction in salt concentration of the solvent was made, based on results comparing 1.6% NaCl and 1.0% NaCl, on three samples (a hard wheat from AACCI Lab Proficiency program check flours, a hard red spring, and a hard red winter flour from the 2020 wheat registration trials). Using this modification, a set of 40 HRS Advanced wheat breeding lines from the 2020 crop year, grown at two locations (Beloeil, QC, and Harrington, PEI, Canada), was tested. The fourth stage involved comparing the performance of flour versus wholemeal using the modified GPT protocol. Samples from the 2020 wheat registration trials in Ontario and Quebec were selected, with one set tested on flour milled by the Bühler MLU-202 mill, and the other on wholemeal produced by a UDY mill. Based on the results, the protocol was adjusted to use wholemeal moving forward, while maintaining other conditions the same.

The following sample sets—drawn from different growing years, trials, and wheat classes—were all tested using wholemeal protocol, and the results were correlated with corresponding quality data: (1) the wheat registration samples from 2021 (23 HRW samples and 48 HRS samples) and 2022 (26 HRW samples and 39 HRS samples). The HRW wheat samples were from Ontario and Quebec trials, while the HRS wheat samples came from Ontario, Quebec, and Maritimes trials. (2) samples from the 2022 Ottawa HRS wheat breeding lines (19 Advanced and 64 Preliminary samples). (3) 18 samples from the 2023 HRW Advanced wheat breeding trials (Ottawa and Woodslee in Ontario, and Beloeil in Quebec).

In 2024, the protocol was modified to use 0.5 M CaCl_2_ in distilled water as the GPT solvent to evaluate its efficiency. Three sample sets were tested: (a) 14 samples from the 2024 HRW Advanced wheat breeding trials (Ottawa and Woodslee, Ontario, Canada); (b) 24 samples from the 2024 HRS Advanced wheat breeding lines in Ottawa (Ontario, Canada); and (c) 23 samples from the 2024 HRW wheat registration trials (11 samples from Ontario and 12 samples from Quebec).

### 2.5. Protein Fractionation (PF) Protocol

A modified protein fractionation (PF) technique [26] was adopted to generate three protein fractions from 20 mg of wholemeal: total protein, total glutenin, and insoluble glutenin. To extract the total protein fraction, 1 mL of 0.6% SDS solution was added to 2 mL microcentrifuge tubes (containing 20 mg of sample) and vortexed at medium speed for 10 s. The tubes were placed in a thermomixer (Eppendorf Thermomixer C, Thermo Fisher, Ottawa, ON, Canada) for 10 min at 25 °C and 1200 RPM, then sonicated using a prob (Fisherbrand™ 120 Sonic Dismembrator with 3 mm diameter probe, Fisher Scientific, Pittsburgh, PA, USA) for 30 s at 20% amplitude, placed back in the thermomixer for another 15 min, and finally centrifuged at 15,000× *g* for 10 min. The supernatant was diluted 55 times (in two steps) with 0.6% SDS solution, and the absorbance was measured at 210 nm using quartz cuvettes (Genesys 10S UV-VIS Spectrophotometer, Thermo Scientific, Pittsburgh, PA, USA). To extract the total glutenin fraction, 1 mL of 7.5% 1-propanol containing 0.3 M NaI was added to each sample (20 mg wholemeal) in 2 mL microcentrifuge tubes, vortexed for 10 s at medium speed, placed in the thermomixer for 15 min (25 °C and 1200 RPM), and centrifuged for 3 min at 1000× *g*. The supernatant (containing monomeric proteins) was discarded, and 1 mL of MilliQ water was added to the pellet, vortexed for 10 s at medium speed, centrifuged at 1000× *g* for 3 min, and the supernatant was decanted. These steps to remove monomeric proteins were repeated one more time. The pellet was then resuspended in 1 mL of 0.6% SDS solution and followed the same steps as total protein extraction, except that the final dilution factor was 35 times (in one step). To extract the insoluble glutenin fraction, 1 mL of 0.6% SDS solution was added to each sample (20 mg wholemeal), vortexed for 10 s at medium speed, placed in the thermomixer for 15 min (25 °C and 1200 RPM), centrifuged for 3 min at 1000× *g*, and the supernatant was discarded. These steps to remove soluble proteins were repeated one more time (the thermomixer step was omitted in the second repeat). The pellet was resuspended in 1 mL of 0.6% SDS solution, and followed the same steps as the total protein extraction, except the final dilution was 20 times (in one step). The dilution factors can be adjusted for specific samples if the absorbance falls outside of the 0.6 to 1.0 AU range. The protein content of each fraction was expressed as AU/mg of wholemeal. Based on these data, the total monomeric protein (total protein—total glutenin) and soluble glutenin (total glutenin—insoluble glutenin) fractions, as well as the percentage and ratio between protein fractions, were calculated.

Five sample sets were tested against the PF protocol, and the results were compared to the corresponding quality traits measured for each sample set. These sets include: (1) 17 samples from the 2021 HRW Advanced wheat breeding trials (Ottawa and Woodslee, Ontario, Canada); (2) 42 samples from the 2023 HRS Advanced wheat breeding trials (Ottawa in Ontario, and Harrington in PEI); (3) 24 samples from the 2024 HRS Advanced wheat breeding, Ottawa, Ontario; (4) 14 samples from the 2024 HRW Advanced wheat breeding trials (Ottawa and Woodslee, Ontario, Canada); and (5) 23 samples from the 2024 wheat registration trials in Ontario and Quebec.

### 2.6. Statistical Analysis

Each test was repeated three or four times, and the results were averaged. Single regression statistical analysis was performed using the Microsoft 365 Excel app (Version 2504, 2024). Multivariate analysis is a powerful and informative tool for visualizing associations among tested parameters, particularly when working with complex datasets. Biplots are widely used in this context, especially in breeding studies, to illustrate such relationships [27]. A two-way data analysis model was selected, using GGEbiplot software (Version 7.10), and genotype-by-trait biplots were generated for each wheat class in each year separately. The biplots were constructed based on the “trait-standardized” GT data, indicated by “Scaling = 1” and “Centering = 2,” and on “trait-focused” singular value partitioning, indicated by “SVP = 2” on the biplot. The biplot origin displays the mean value for each trait. All biplot graphs show the relationships among testers (the parameters included in the statistical analysis), with angles less than 90 degrees indicating stronger positive associations, angles greater than 90 degrees indicating negative associations, and right angles indicating no significant association. Longer vectors (parameters farther from the biplot origin) denote greater discrimination among tested genotypes. Regression values and their significance levels are available in the supplemental tables. The correlation coefficients presented in the supplementary tables serve as additional validation of the patterns observed in the biplots. However, to highlight the most statistically significant associations (*p* < 0.05), relevant correlation values have been directly incorporated into the main text where appropriate.

## 3. Results

### 3.1. GPT Protocol Development and Testing

The initial testing aimed to identify a method that effectively distinguishes gluten aggregation behavior across a diverse range of flours. Based on the literature reports, six methods (Table 2) were tested on three types of hard wheat flours and one sample of soft wheat flour; the results are shown in Table 3. The GlutoPeak Tester (GPT) software (GlutoPeak^®^, version 2.2.10) automatically calculates parameters such as PMT, BEM, AM, PM, A01, A12, A23, A34, and A45. BEM is the maximum torque value and indicates gluten strength. As expected, all methods except Method 2 displayed significantly lower BEM for the soft wheat flour sample compared to the other three hard wheat flour samples. In Method 2, the solvent amount was adjusted based on the flour’s moisture and protein content, which might explain the different ranking observed. Method 3 produced the highest BEM values for all the flour samples, likely due to a higher amount of flour used. The time of maximum torque (PMT) varied significantly among the different methods, ranging from 56.7 s to 377. 3 s, probably due to variations in solvent types and speed (RPM) used in these methods, especially Methods 5 and 6, which had distilled water as the GPT solvent, resulting in a longer time for the torque to peak. Conversely, a higher salt concentration (2% *w*/*v*) and increased RPM in Method 4 reduced the differences in PMT among the different flour samples. All methods showed the highest aggregation energy (A34) for flour 2, which was the strongest flour. The gluten strength index (GSI) values, calculated as BEM × total area [25], clearly distinguished hard flour samples from soft flour. Based on these results, the parameters of Method 1 were selected for the next phase.

**Figure 1:**
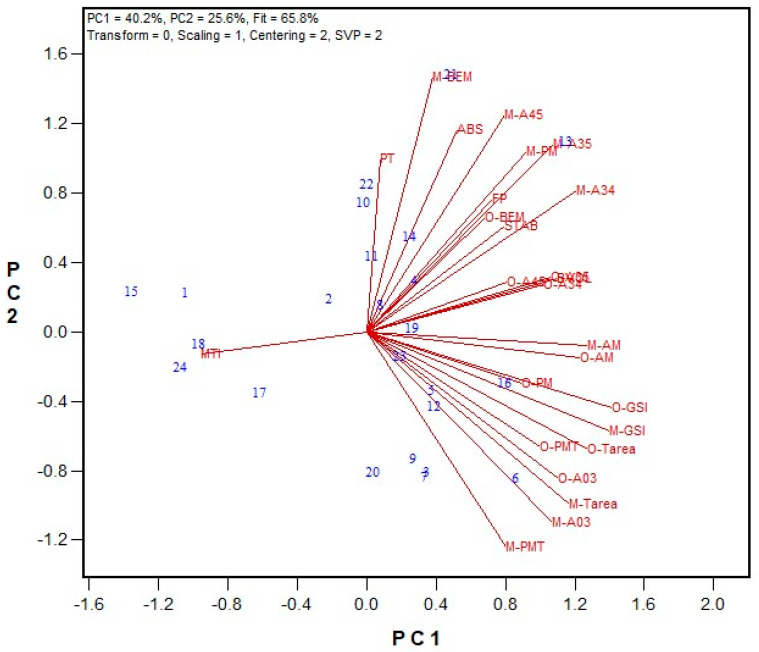
Biplot illustrating relationships among parameters tested for the 2018 HRW wheat registration flour samples in the Ottawa and Moulins labs. Abbreviations: GPT parameters (PMT, BEM, AM, PM, A03, A34, A35, A45, total area/TArea, GSI) are from the Ottawa lab (O-) and Moulins lab (M-); flour protein (FP); Farinograph parameters, including water absorption (ABS), peak time (PT), stability (STAB), and mixing tolerance index (MTI); bread volume (BVOL). Numbers represent tested genotypes (n = 24).

In the second stage, the performance of GPT machines in two laboratories was evaluated using Method 1 parameters. Both labs analyzed the same flour samples, which were milled from wheat grains obtained from the 2018 wheat registration trials in Ontario and Quebec. Additionally, data on flour protein content, Farinograph parameters, and bread volume were collected. Among the 24 hard red winter (HRW) wheat samples tested, flour protein content ranged from 9.6% to 12.3% (on a 14% moisture basis), and Farinograph ABS varied between 55.8% and 66.9%. Bread volume ranged from 524 cm^3^ to 703 cm^3^. Regarding the GPT results, PMT values were consistently higher for the Moulins unit, ranging from 74 to 262 s (average 146 s), compared to the Ottawa unit, which ranged from 24 to 90 s (average 43 s). Meanwhile, BEM, AM, and PM values were slightly higher for the Ottawa unit than for the Moulins unit. These opposing effects led to slightly higher A34, A45, and A35 values for the Ottawa unit, whereas the Moulins unit exhibited significantly higher A03, total area, and GSI values. Biplots were generated to compare the overall rankings between the two labs and to examine correlations with Farinograph and bread baking results. The biplot in Figure 1 illustrates the relationships among the tested traits for the HRW wheat samples, while Supplemental Table S1 provides the regression values. The PC1 and PC2 in the biplot explained 65.8% of the observed associations. Most GPT parameters showed statistically significant correlations between the two machines (except for BEM, PM, and A45). The strongest correlations were observed for total area (r = 0.855), A03 (r = 0.862), and the GSI (r = 0.865). Notably, BEM was not significantly correlated between the two GPT machines (r = 0.380), indicating that flour gluten strength was not ranked the same in both units based on BEM. However, the GSI (total area × BEM) ranked wheat flour strengths similarly across both labs. Several significant correlations between measured quality parameters and GPT results were identified. For the Ottawa unit, bread volume correlated with the GSI (r = 0.490) and PMT (r = 0.527), while for the Moulins unit, A34 significantly correlated with most quality traits, including Farinograph absorption (ABS, r = 0.656) and stability (STAB, r = 0.525), as well as bread volume (r = 0.639). Additionally, most GPT parameters from the Moulins unit showed significant correlations with Farinograph ABS. 

**Figure 2:**
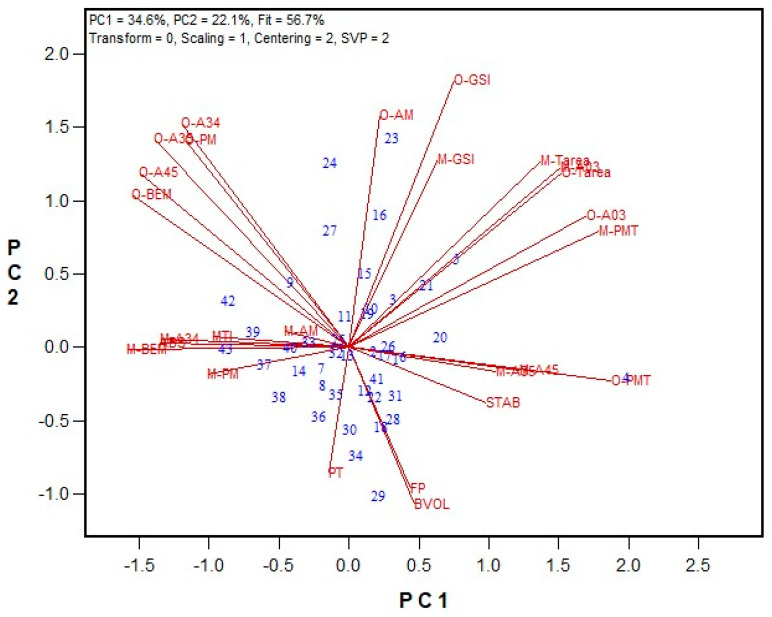
The biplot illustrating relationships among parameters tested for the 2018 HRS wheat registration flour samples in the Ottawa and Moulins labs. Abbreviations: GPT parameters (PMT, BEM, AM, PM, A03, A34, A35, A45, total area/TArea, GSI) are from the Ottawa lab (O−) and Moulins lab (M−); flour protein (FP); Farinograph parameters, including water absorption (ABS), peak time (PT), stability (STAB), and mixing tolerance index (MTI); bread volume (BVOL). Numbers represent tested genotypes (n = 43).

For the hard red spring (HRS) wheat class, 43 flour samples were tested by both labs. A wide range of flour protein content (12.2–16.5% on a 14% moisture basis) were observed, while the Farinograph ABS ranged from 55.9% to 64.5%, and bread volume varied between 693 cm^3^ and 964 cm^3^. Figure 2 shows a biplot comparing results between the Ottawa and Moulins labs, with regression values listed in Supplemental Table S2. The biplot fit, measured by PC1 and PC2, was 56.7%, slightly lower than that for the HRW wheat class. The PMT values between the Ottawa and Moulins units were closer (average 111 s and 152 s, respectively) than those of the HRW wheat class. However, A03, total area, and GSI values were higher for the Moulins unit. In the HRS wheat class, the vectors were shorter than those of the HRW, indicating weaker correlations among traits. Similarly to the HRW wheat results, the HRS results showed significant correlations (*p* < 0.05) between GPT parameters in the Ottawa and Moulins labs, including the GSI (r = 0.719), total area (r = 0.876), and A03 (r = 0.886). Farinograph ABS displayed higher correlations with several GPT data for the Moulins unit (PMT, r = −0.741; BEM, r = 0.855; A34, r = 0.814) compared to the Ottawa unit (PMT, r = −0.337; BEM, r = 0.370; A34, r = 0.412). Farinograph STAB were significantly correlated with A03 (r = 0.325), and PMT (r = −0.444) for the Ottawa unit, and PMT (r = 0.351) for the Moulins unit. Farinograph peak time (PT) were significantly correlated with PMT (r = −0.302), BEM (r = 0.336), AM (r = 0.391), and PM (r = 0.392) for the Moulins unit, but not with any GPT parameters in the Ottawa lab. Interestingly, among Farinograph data, PT was the only parameter correlated significantly with bread volume (r = 0.596).

**Figure 3:**
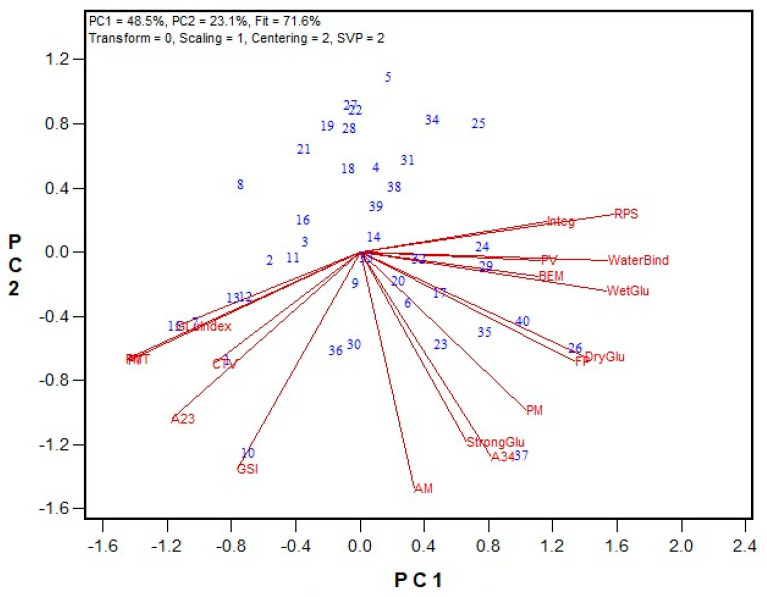
Biplot illustrating relationships among parameters tested for the 2020 HRS Advanced wheat breeding lines. Abbreviations: GPT parameters, including PMT, BEM, AM, PM, A23, A34, and GSI; flour protein (FP); Mixograph parameters include peak time (PT), peak value (PV), right of peak slope (RPS), curve tail value (CTV), and integral at the end of the run (Integ); Glutomatic parameters include wet gluten (WetGlu), gluten index (GluIndex), dry gluten (DryGlu), water binging capacity (WaterBind), and strong gluten index (StrongGlu). Numbers represent tested genotypes (n = 40).

In the third testing stage, the reduction in salt concentration, from 1.2% to 1.0%, was examined across three hard wheat flour samples. Both 1.6% and 1% NaCl solvents rated the flours similarly across all GPT parameters; the AACCI check flour showed the highest BEM, while the HRS wheat had the longest PMT and the highest GSI (Table 4). Based on these results, NaCl content was reduced to 1% (*w*/*v*). Using this adjustment, a set of 40 HRS wheat flour samples (from the 2020 Advanced breeding lines) was tested, and the results were compared with Mixograph and Glutomatic data (Figure 3 and Supplemental Table S3). Flour protein content in this set ranged from 12.6% to 16.9% (on a 14% moisture basis). Mixograph peak values varied between 41.3% to 55.9%, while Glutomatic gluten indices ranged between 80.2% to 99.5%. Several GPT parameters showed significant correlations with Mixograph and Glutomatic data. The PMT and Mixograph peak time demonstrated a very strong regression (r = 0.900), while its correlation with the peak value was negative (r = −0.523). A negative correlation was also observed between PMT and Glutomatic wet gluten content (r = −0.661), dry gluten content (r = −0.494), and water binding capacity (r = −0.713). Similarly, BEM showed significant correlations with most Mixograph and Glutomatic data, with Mixograph peak time (r = −0.530) and integral at the end of the run (r = 0.511) presenting the strongest r values. The AM and PM results were both significantly correlated with the Glutomatic strong gluten index (r = 0.624 and 0.518, respectively). Energy values of A23 also had significant correlations with most quality data, with Mixograph peak time (r = 0.842) and the Glutomatic gluten index (r = 0.560) presenting the highest values. The associations between the GSI values and Mixograph curve tail value (r = 0.493) and the Glutomatic gluten index (r = 0.461) were also statistically significant (*p* < 0.05).

**Figure 4:**
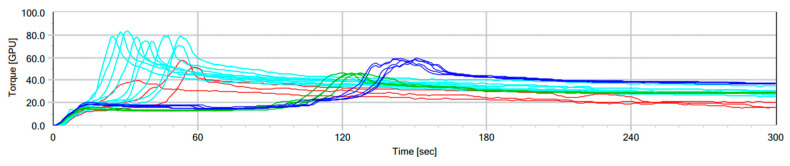
Overlayed GPT graphs of wheat flour and wholemeal samples. Samples—from the 2020 Ontario and Quebec wheat registration trials—are HRS wheat flour, dark blue lines; HRW wheat flours, green lines; HRS wholemeal wheat, light blue lines; and HRW wholemeal wheat, red lines.

In the fourth stage, the method was adjusted to use wholemeal instead of refined wheat flour. Samples from the 2020 wheat registration trials in Ontario and Quebec were tested on both flour and wholemeal. Figure 4 shows the GPT graphs of flour samples and wholemeal samples from both the HRS and HRW wheat classes. A clear distinction appeared between the HRS and HRW wheat flour samples, with HRS showing higher torque (BEM) and longer PMT values. Meanwhile, the wholemeal samples exhibited a significant reduction in PMT compared to their respective flour samples, facilitating a shorter run time. The HRS wheat wholemeal samples had significantly higher BEM values than their corresponding wheat flour samples, although this trend was less pronounced in the HRW wheat class.

**Figure 5:**
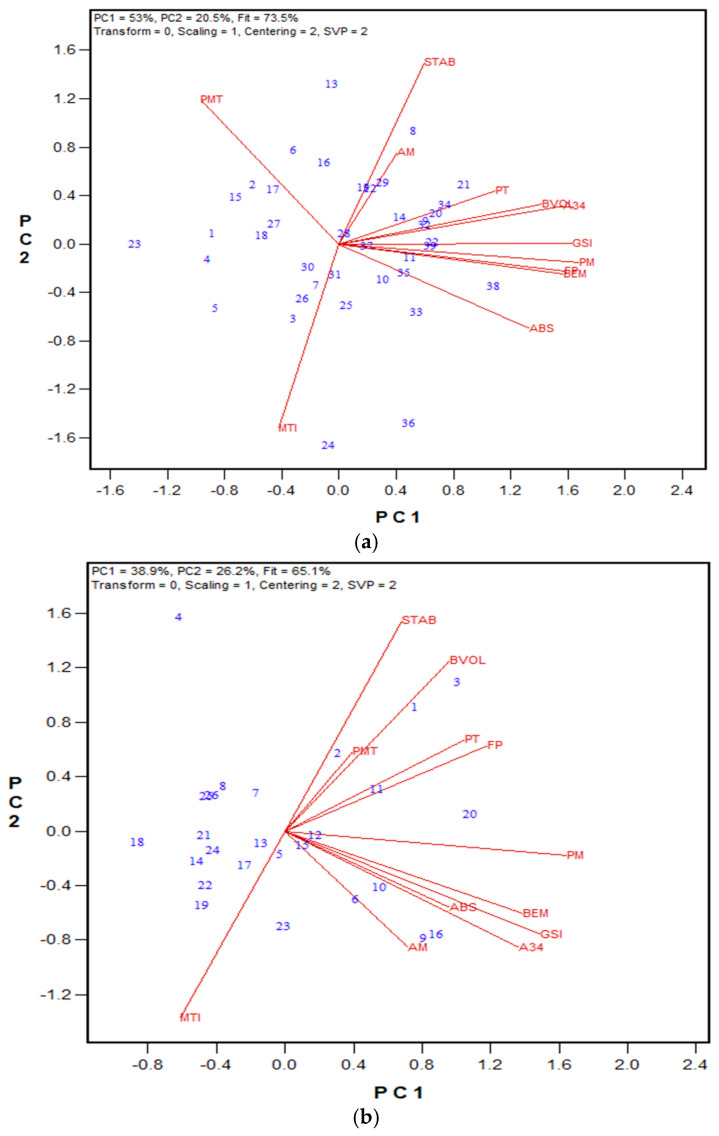
Biplot illustrating relationships among parameters tested for (**a**) 48 HRS and (**b**) 23 HRW samples, both from the 2021 wheat registration trials. Abbreviations: GPT parameters, including PMT, BEM, AM, PM, A34, and GSI; flour protein (FP); Farinograph parameters, including water absorption (ABS), peak time (PT), stability (STAB), and mixing tolerance index (MTI); bread volume (BVOL). Numbers represent tested genotypes.

**Figure 6:**
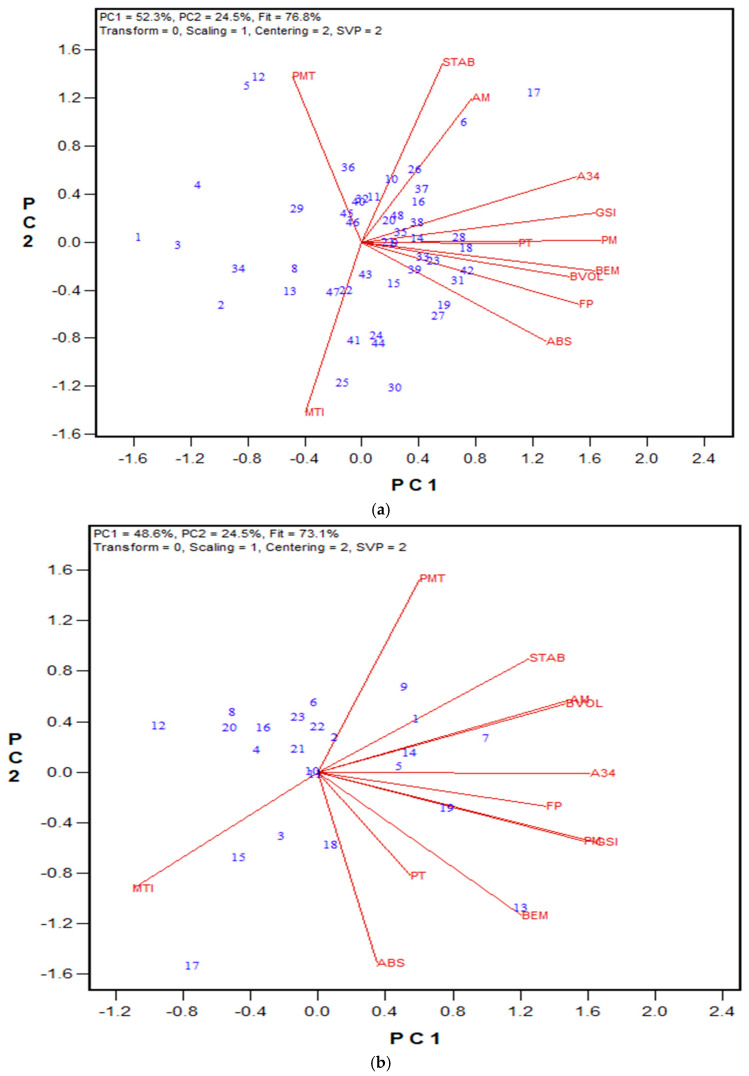
Biplots illustrating relationships among parameters tested for (**a**) 39 HRS and (**b**) 26 HRW samples, both from the 2022 wheat registration trials. Abbreviations: GPT parameters, including PMT, BEM, AM, PM, A34, and GSI; flour protein (FP); Farinograph parameters, including water absorption (ABS), peak time (PT), stability (STAB), and mixing tolerance index (MTI); bread volume (BVOL). Numbers represent tested genotypes.

**Figure 7:**
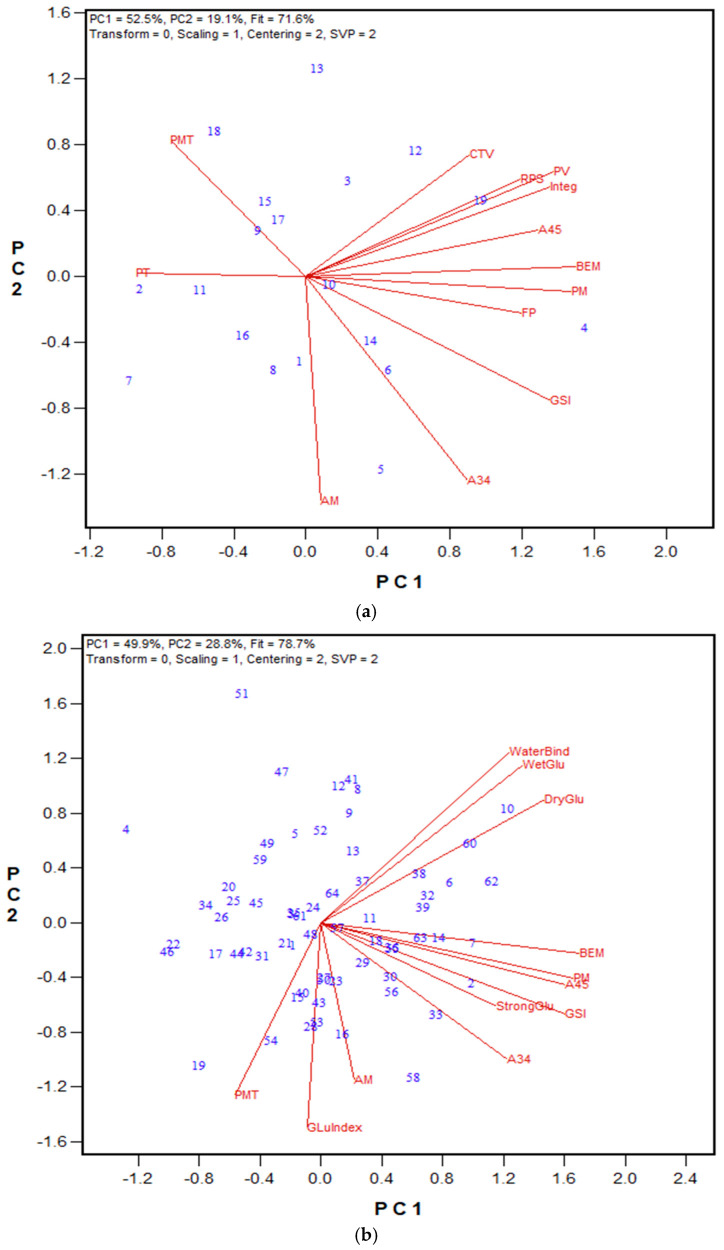
Biplots illustrating relationships among parameters tested for (**a**) 19 HRS Advanced and (**b**) 64 HRS Preliminary breeding lines, both from the 2022 HRS wheat breeding trials in Ottawa (Ontario, Canada). Abbreviations: GPT parameters, including PMT, BEM, AM, PM, A34, A45, and GSI; flour protein (FP); Mixograph parameters, including peak time (PT), peak value (PV), right of peak slope (RPS), curve tail value (CTV), and integral at the end of the run (Integ); Glutomatic parameters, including wet gluten (WetGlu), gluten index (GluIndex), dry gluten (DryGlu), water binging capacity (WaterBind), and strong gluten index (StrongGlu). Numbers represent tested genotypes.

**Figure 8:**
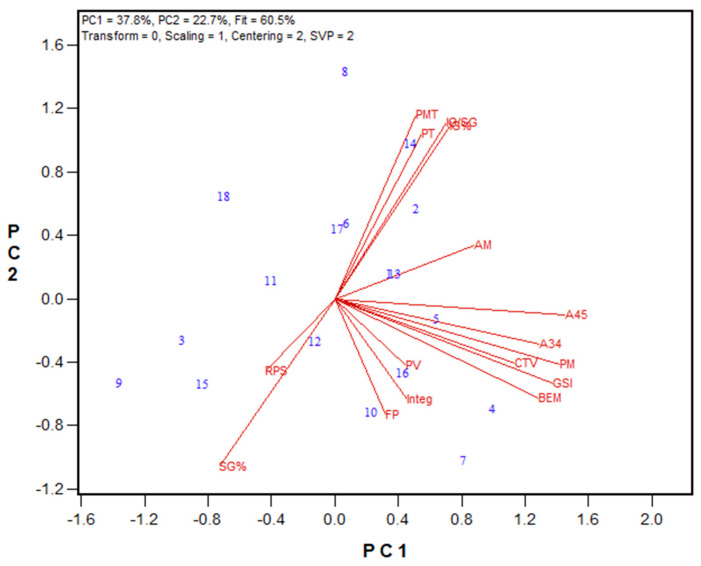
Biplot illustrating relationships among parameters tested for the 2023 HRW Advanced breeding lines from three locations (Ottawa and Woodslee in Ontario, and Beloeil in Quebec, Canada). Abbreviations: GPT parameters, including PMT, BEM, AM, PM, A34, A45, and GSI; flour protein (FP); Mixograph parameters, including peak time (PT), peak value (PV), right of peak slope (RPS), curve tail value (CTV), and integral at the end of the run (Integ); soluble glutenin% (SG%); insoluble glutenin% (IG%); the ratio of insoluble to soluble glutenin (IG/SG). Numbers represent tested genotypes (n = 18).

To evaluate the wholemeal protocol across different sample sets, growing seasons, and wheat classes, the following tests were performed: (1) the 2021 and 2022 wheat registration samples (both HRW and HRS wheat); (2) two sets of 2022 HRS breeding lines from the Ottawa location; and (3) a set of 2023 HRW advanced breeding lines from three locations (Ottawa, Woodslee, and Beloeil). Figure 5, Figure 6, Figure 7 and Figure 8 show the biplots of the relationships between the GPT parameters and other quality data, with regression values provided in Supplemental Tables S4–S10.

For the HRS wheat registration sets from both 2021 and 2022, A34 showed significant correlations with Farinograph ABS, PT, and STAB, as well as bread volume (Figure 5A and Figure 6A). Farinograph ABS was also negatively associated with PMT and positively associated with the GSI in both years. Strong correlations were observed between BEM and bread volume (r = 0.749 and 0.631 in 2021 and 2022, respectively), and consequently between the GSI and bread volume (r = 0.664 and 0.683 in 2021 and 2022, respectively). Regarding the HRW wheat class, similar trends in associations were observed between 2021 and 2022 for PMT and Farinograph ABS (negative regressions) and STAB (positive regressions), as shown in Figure 5B and Figure 6B. In 2021, several GPT parameters were also linked to Farinograph parameters and bread volume, with BEM and Farinograph ABS showing the strongest regression (r = 0.653). We proposed a new GPT parameter, which calculates the difference between PM and AM (PM-AM), to explore changes between the degree of gluten breakdown (PM, 15 s after the peak) and the initial aggregation point (AM, 15 s before the peak), reflecting the resistance of aggregated gluten to shearing forces. PM-AM values showed significant correlations with bread volume, Farinograph ABS, and PT for the HRS sample sets (r = 0.653, 0.649, and 0.554, respectively, *p* < 0.0001, for the 2021 sample set) and (r = 0.513, *p* = 0.0008; r = 0.609, *p* < 0.0001; and r = 0.472, *p* = 0.002, respectively, for the 2022 sample set). For the HRW sample sets, Farinograph ABS was significantly correlated with PM-AM values (r = 0.689, *p* = 0.0003, for the 2021 sample set).

Among tested HRS and HRW Advanced breeding lines, PMT and Mixograph peak time were strongly correlated for both wheat classes (r = 0.660 and 0.819 for HRS trials in 2022 and HRW trials in 2023, respectively). Additionally, BEM and Mixograph curve tail value were significantly associated in both wheat classes. Overall, more associations were observed in the HRS wheat class compared to the HRW wheat class (Figure 7A and Figure 8). Interestingly, for the 2022 HRS Preliminary breeding lines, most GPT parameters showed significant associations with Glutomatic results, with the strongest regressions observed for PMT and the gluten index (r = 0.668), BEM and dry gluten content (r = 0.645), and the GSI and strong gluten content (r = 0.563). Similar to the wheat registration sample sets, PM-AM values were calculated and linked to quality parameters. For Advanced lines in both the HRW and HRS classes, several Mixograph parameters were associated with PM-AM values, including peak value, right of peak slope, and integral at the end of run (r = 0.646, 0.650, and 0.585, respectively, *p* < 0.001) in the 2022 HRS trials, and curve tail value (r = 0.475, *p* < 0.05) in the 2023 HRW trials. Like other GPT parameters, PM-AM showed significant correlation (*p* < 0.0001) with Glutomatic results in the 2022 HRS Preliminary trials, including dry gluten content, wet gluten content, and water binding capacity (r = 0.633, 0.603, and 0.579, respectively).

**Figure 9:**
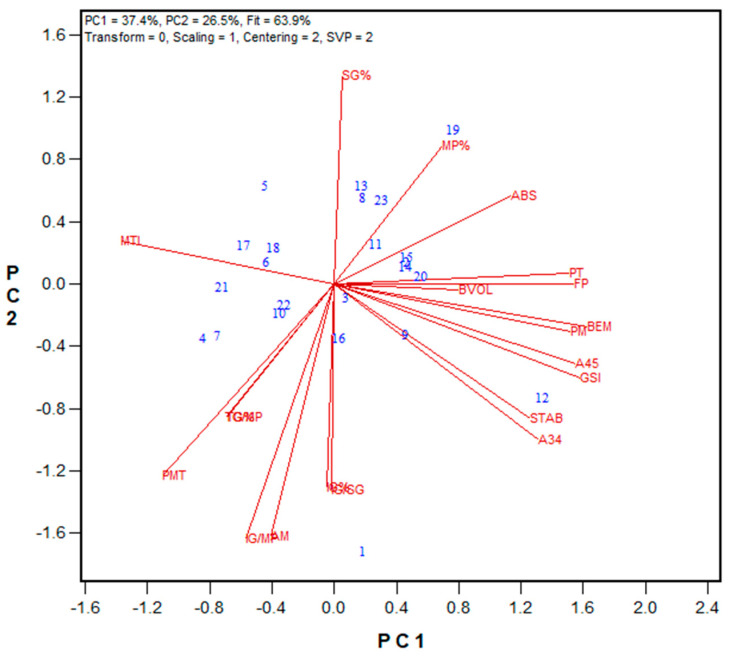
Biplot illustrating relationships among parameters tested for the 2024 HRW wheat registration wholemeal samples from Ontario (n = 11) and Quebec (n = 12) trials, Canada. Abbreviations: GPT parameters, including PMT, BEM, AM, PM, A34, A45, and GSI; flour protein (FP); Farinograph parameters, including water absorption (ABS), peak time (PT), stability (STAB), and mixing tolerance index (MTI); bread volume (BVOL); total glutenin% (TG%); insoluble glutenin fraction% (IG%); soluble glutenin fraction% (SG%); monomeric protein% (MP%); the ratio of insoluble to soluble glutenin (IG/SG); the ratio of insoluble glutenin to monomeric protein (IG/MP). Numbers represent tested genotypes.

**Figure 10:**
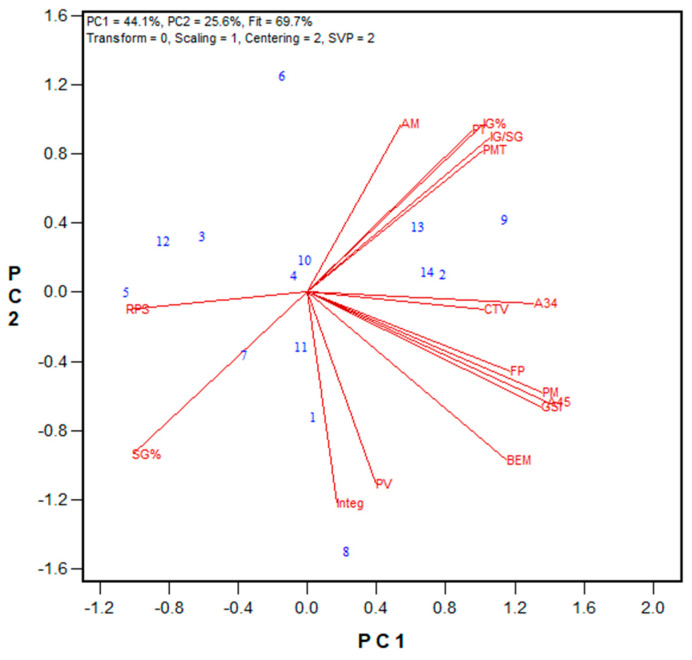
Biplot illustrating relationships among parameters tested for the 2024 HRW Advanced wheat breeding lines from two locations (Ottawa and Woodslee, Ontario, Canada). Abbreviations: GPT parameters, including PMT, BEM, AM, PM, A34, A45, and GSI; flour protein (FP); Mixograph parameters, including peak time (PT), peak value (PV), right of peak slope (RPS), curve tail value (CTV), and integral at the end of the run (Integ); Soluble glutenin% (SG%); insoluble glutenin% (IG%); the ratio of insoluble to soluble glutenin (IG/SG). Numbers represent tested genotypes (n = 14).

**Figure 11:**
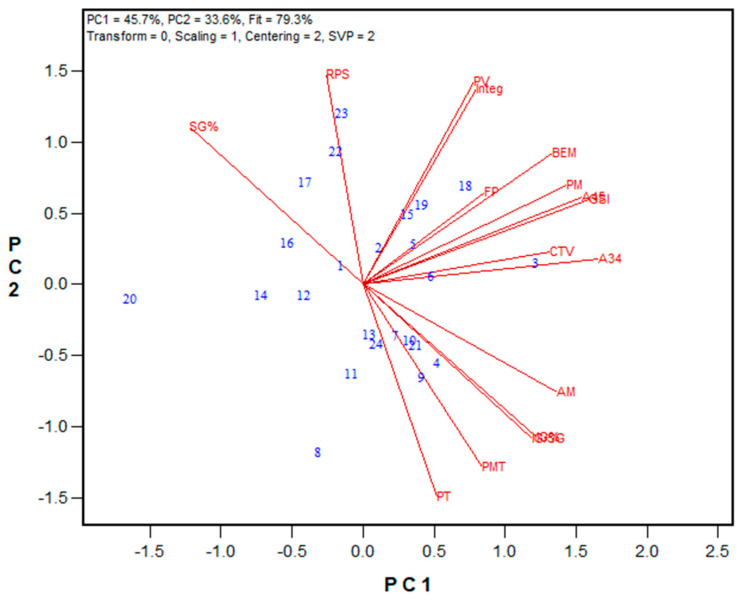
Biplot illustrating relationships among parameters tested for the 2024 HRS Advanced wheat breeding lines from Ottawa (Ontario, Canada). Abbreviations: GPT parameters, including PMT, BEM, AM, PM, A34, A45, and GSI; flour protein (FP); Mixograph parameters, including peak time (PT), peak value (PV), right of peak slope (RPS), curve tail value (CTV), and integral at the end of the run (Integ); Soluble glutenin% (SG%); insoluble glutenin% (IG%); the ratio of insoluble to soluble glutenin (IG/SG). Numbers represent tested genotypes (n = 24).

In 2024, the GPT protocol was updated by replacing the 1% NaCl with a 0.5 M CaCl_2_ solution. Stronger salt solution caused significant dropping in PMT values for both the HRS and HRW wheat classes. Despite this change, all other GPT parameters remained largely unchanged. Notably, the method’s repeatability improved, especially for PMT and BEM values (Table 5). The revised protocol was tested with three independent sample sets. Figure 9, Figure 10 and Figure 11 display biplots showing the relationships between GPT parameters and other quality data, while the regression results are listed in Supplemental Tables S11–S13. For the 2024 HRS Advanced breeding lines, the Mixograph curve tail value showed significant correlation with most GPT results, including A34, GSI, BEM, and PMT (r = 0.672, 0.667, 0.585, and 0.519, respectively). The strongest correlation was between PMT and Mixograph peak time (r = 0.874). Concerning the 2024 HRW class, both the wheat registration set and the Advanced breeding lines displayed significant correlations with several other quality metrics. Similarly to the HRS class, in the 2024 HRW Advanced breeding lines, the Mixograph curve tail value correlated with A34 and PMT (r = 0.594 and 0.562, respectively). Again, the highest correlation was observed between PMT and Mixograph PT (r = 0.770). In the 2024 HRW wheat registration set, the Farinograph PT and STAB correlated with BEM (r = 0.649 and 0.654), A34 (r = 0.594 and 0.657), and the GSI (r = 0.680 and 0.696). PM-AM values showed significant correlations with Farinograph PT and ABS (r = 0.610 and 0.735, respectively, *p* < 0.001) for the 2024 HRW wheat registration set, and with Mixograph peak time, peak value, right of peak slope, and integral at the end of run (r = 0.639, 0.563, 0.563, and 0.560, respectively, *p* < 0.01) for the 2024 HRS Advanced breeding lines.

### 3.2. Protein Fractionation Protocol Testing

**Figure 12:**
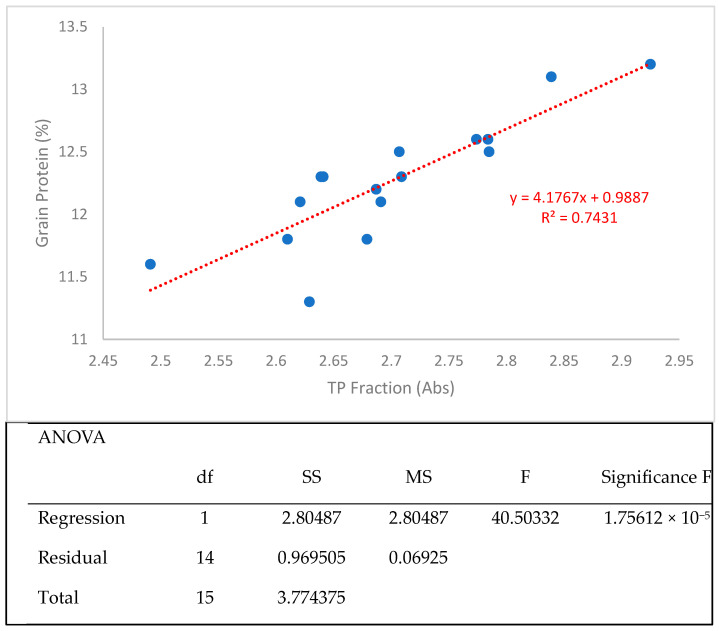
Regression curve and its ANOVA results illustrating the correlation between grain protein and wholemeal TP fraction content of the 2021 HRW Advanced wheat breeding lines (n = 17, each with four repeats) from Ottawa and Woodslee (Ontario, Canada).

Since the original method reported by Fu et al. [26] was developed for the HRS wheat class, an initial attempt was made to assess whether this method could also differentiate samples from the HRW wheat class. A strong and statistically significant correlation was observed between grain protein% (measured by an NIRS technique) and the TP fraction (absorbance values) in the 2021 HRW Advanced wheat breeding lines, validating the accuracy of the PF method in ranking genotypes based on total protein content (Figure 12).

**Figure 13:**
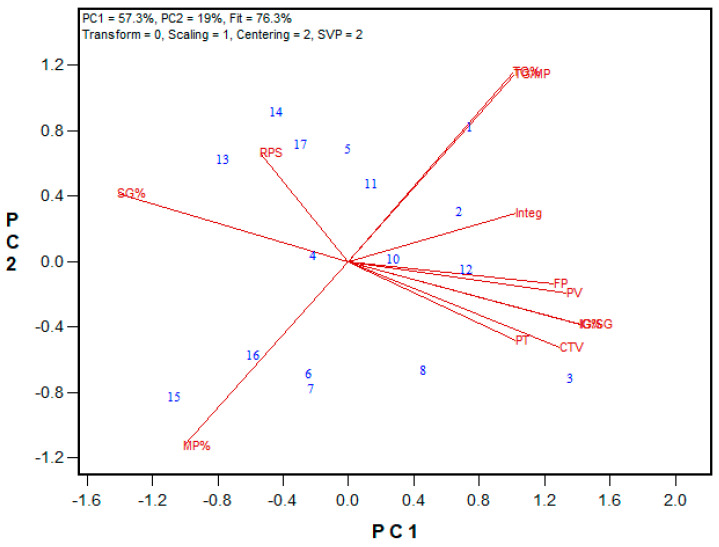
Biplot illustrating relationships among parameters tested for the 2021 HRW Advanced wheat breeding lines from Ottawa and Woodslee (Ontario, Canada). Abbreviations: flour protein (FP); Mixograph parameters, including peak time (PT), peak value (PV), right of peak slope (RPS), curve tail value (CTV), and integral at the end of the run (Integ); total glutenin% (TG%); insoluble glutenin fraction% (IG%); soluble glutenin fraction% (SG%); monomeric protein% (MP%); the ratio of insoluble to soluble glutenin (IG/SG); the ratio of insoluble glutenin to monomeric protein (IG/MP). Numbers represent tested genotypes (n = 17).

Figure 13 illustrates the biplot of the 2021 HRW Advanced breeding lines results, and the regression values are presented in Supplemental Table S14. The genotypes showed a wide range of insoluble glutenin% (26.5% to 56.6%), with Woodslee location showing lower values compared to their relevant genotypes in the Ottawa location, indicating the environmental effect on the gluten profile. The strongest correlations were found between Mixograph parameters and insoluble glutenin% and the ratio of insoluble to soluble glutenin. These include peak time (r = 0.732 and 0.739), peak value (r = 0.752 and 0.767), and curve tail value (r = 0.766 and 0.813) associations with insoluble glutenin% and the ratio of insoluble to soluble glutenin, respectively.

**Figure 14:**
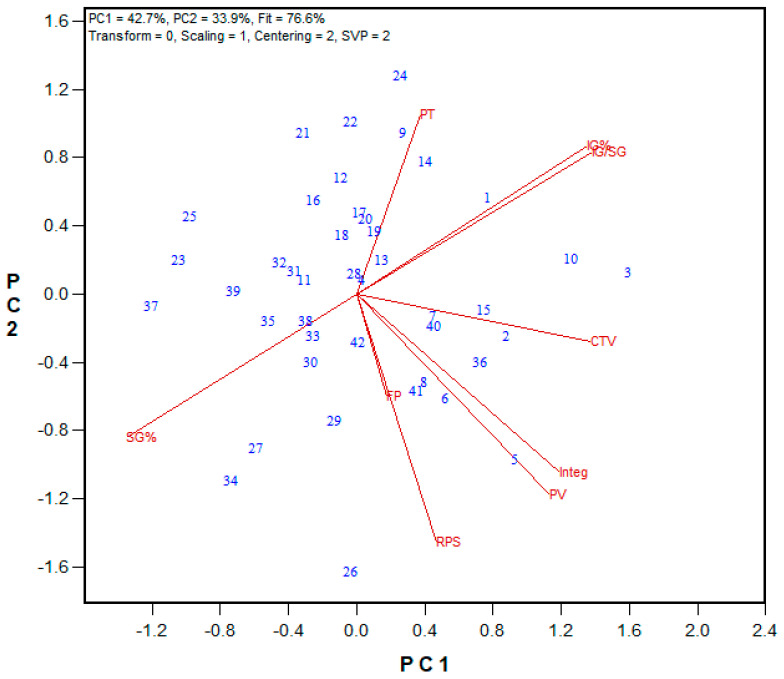
Biplot illustrating relationships among parameters tested for the 2023 HRS Advanced wheat breeding lines from Ottawa, Ontario, and Harrington, PEI. Abbreviations: flour protein (FP); Mixograph parameters, including peak time (PT), peak value (PV), right of peak slope (RPS), curve tail value (CTV), and integral at the end of the run (Integ); Soluble glutenin% (SG%); insoluble glutenin% (IG%); the ratio of insoluble to soluble glutenin (IG/SG). Numbers represent tested genotypes (n = 42).

Based on these results, only the total glutenin and insoluble glutenin fractions were extracted for the 2023 and 2024 wheat breeding sets. The corresponding biplot results are shown in Figure 8, Figure 10, Figure 11 and Figure 14, while regression values are provided in Supplemental Tables S10, S12, S13 and S15. Regardless of the harvest year and sample set size, the insoluble glutenin% of HRW wheat ranged from 26% to 57%, with an average of 42%, while HRS wheat showed a broader range from 18% to 52%, with an average of 39%. The ratio of insoluble glutenin to soluble glutenin was as low as 0.23 and 0.34, and as high as 1.0 and 1.4 for HRS and HRW wheat, respectively. Similarly to the 2021 HRW set, the 2023 HRS Advanced wheat breeding lines demonstrated significant associations between the Mixograph curve tail value and peak time with insoluble glutenin% (r = 0.452 and 0.345, respectively) and the ratio of insoluble to soluble glutenin (r = 0.486 and 0.363, respectively), as shown in Figure 14. These associations were even stronger in the 2024 HRS Advanced lines (Figure 11), with correlation coefficients of 0.644 and 0.634 between Mixograph peak time and insoluble glutenin% and the insoluble to soluble glutenin ratio, respectively. Additionally, the right of the peak slope parameter displayed significant negative correlations with insoluble glutenin% (r = −0.521) and the ratio of insoluble to soluble glutenin (r = −0.503), indicating that stronger gluten in the flour resulted in less dough breakdown during overmixing. Both insoluble glutenin% and the ratio of insoluble to soluble glutenin had statistically significant correlations with several GPT parameters in this set. The strongest correlations were observed with AM (r = 0.765 and 0.756, respectively) and PMT (r = 0.699 and 0.684, respectively). For the 2024 HRW Advanced wheat breeding lines, both PMT and Mixograph peak time were correlated with insoluble glutenin% (r = 0.606 and 0.554) and the ratio of insoluble to soluble glutenin (r = 0.576 and 0.541), respectively (Figure 10).

To assess the relationship between PF results and Farinograph parameters, the 2024 HRW wheat registration sample set was tested using the PF protocol, with all three fractions extracted (Figure 9). While PF results did not show significant correlations with Farinograph parameters (Supplemental Table S11), they did exhibit notable associations with GPT parameters. Specifically, PMT and AM were correlated with insoluble glutenin% (r = 0.589 and 0.613), the ratio of insoluble to soluble glutenin (r = 0.591 and 0.623), the ratio of insoluble glutenin to monomeric protein (r = 0.732 and 0.814), and the ratio of total glutenin to monomeric protein (r = 0.419 and 0.459), respectively.

## 4. Discussion

The potential of the GlutoPeak Tester (GPT) technique as a tool for assessing gluten aggregation behavior has been supported by numerous publications over the past decade, with a wide range of applications [5,6,7,12,14,15,17,20,28,29]. While GPT has been shown to predict various quality traits, discussions continue about which specific traits it predicts most effectively and through which GPT parameter. A comparative study [25] evaluating GPT performance across two distinct sets of wheat samples—one with similar Farinograph absorption (ABS) values but differing gluten strength, and another with consistent gluten strength but a wide range of ABS—highlighted the limitations of using PMT or BEM alone to rank samples. The findings indicated that the gluten strength index (GSI, calculated as BEM × total area) serves as a more reliable parameter for comparing gluten aggregation behavior, particularly in diverse wheat sample sets. In our study, the GSI was consistently calculated to evaluate its effectiveness in discriminating among wheat genotypes in eastern Canada environments. However, due to inconsistencies in the GPT software’s ability to accurately record certain energy values (A01, A12, A23, A34, and A45), different formulas were applied depending on whether flour or wholemeal samples were analyzed. These methodological adjustments are elaborated in the relevant sections of the paper.

To the best of our knowledge, there is no report in the literature about comparing the performance of GPT across different labs. The comparison testing performed in our study showed that, despite some differences between the GPT units in the Ottawa and Moulins labs, the overall genotype rankings for both hard red spring (HRS) and hard red winter (HRW) wheat classes were similar in both labs when using the same materials and method during the second phase of testing. This similarity was particularly evident for the GSI parameter, calculated as BEM × total area. A ring study of this nature, involving multiple users to compare GPT performance, would be highly beneficial for establishing a more standardized methodology and facilitating consistent evaluation of results. Among GPT parameters, the GSI was positively associated with bread volume in the HRW wheat class in both labs, whereas PMT showed a stronger correlation with bread volume in the HRS class (only in the Ottawa lab). These findings suggest that the predictive utility of GPT may be influenced by wheat class, likely due to differences in gluten composition and protein profiles. This observation aligns with the previous literature reporting substantial variability in GPT results when different wheat classes are analyzed together against other quality metrics [24]. In our study, wheat classes were evaluated separately throughout the protocol development and performance assessment to account for these class-specific differences.

Modifying the NaCl concentration in the GPT solvent during the third testing stage did not result in statistically significant changes in GPT parameters. However, the most noticeable—albeit not statistically significant—effect was observed in the A23 value, which consequently influenced the GSI, calculated as BEM × (A23 + A34). The A23 parameter represents the equilibrium energy during gluten aggregation, and lower NaCl concentrations appeared to slow the equilibration process, likely due to reduced efficiency in water absorption and gluten network formation. Supporting our findings, Amoriello and Carcea [6] evaluated 45 cultivars with and without 1.5% NaCl and reported slight differences in GPT parameters, noting cultivar-dependent behavior. Stronger flours tended to hydrate more slowly, resulting in higher PMT and BEM values. Similarly, Bouachra et al. [7] found that a 1% NaCl solution was more effective than 5% lactic acid and other solvents in eliciting significant GPT responses, particularly in differentiating bread volume among winter wheat varieties grown in diverse regions.

A strong association between PMT and Mixograph peak time observed in the 2020 HRS Advanced breeding lines under reduced salt conditions highlights the potential of GPT as a reliable screening tool for breeding material. This supports the idea that GPT could serve as a viable alternative to the Mixograph, a widely recognized and accepted technique in wheat breeding quality evaluation. Consistent with our findings, a strong correlation between PMT and Mixograph peak time has been reported in previous studies (e.g., r = 0.90 [16]; r = 0.77 [30]), although these results were obtained under different GPT conditions and with wheat flours of varying compositions. Another notable relationship observed in our study was the negative correlation between PMT and Glutomatic wet gluten content, supporting the concept that higher gluten content facilitates earlier protein aggregation. Additionally, BEM exhibited a moderate positive correlation with Mixograph integral at the end of the run, as both parameters reflect the overall strength of the gluten network in the flour. All ten quality traits measured by Mixograph and Glutomatic, for the 2020 HRS Advanced breeding lines, were predicted by at least one and in some cases by five GPT parameters. Supporting our findings and using a multivariate approach similar to our GGEbiplot analysis, Marti et al. [24], based on a study of 120 wheat flour samples, reported that GPT could reliably predict more than half of the quality parameters typically measured by conventional techniques such as the Farinograph, Alveograph, and Extensograph. Similarly, significant correlations were reported [13] for winter bread wheat varieties with different baking quality, with GPT BEM showing correlation coefficients of 0.84, 0.68, and 0.61 (*p* < 0.01) with Farinograph absorption (ABS), Glutomatic wet gluten content, and bread volume, respectively. Using a broad range of bread wheat varieties from three different harvest year [7], a strong correlation (r = 0.77) was also observed between GPT AM and bread volume. Fu et al. [31] found a strong correlation (r = 0.97) between Farinograph ABS and GPT BEM in a dataset of 83 wheat flour samples milled using a Bühler mill, representing various wheat classes. This correlation remained robust (r = 0.96) when tested on 63 flour samples milled with a Quadrumat Junior mill, leading to propose incorporation of GPT as a rapid screening method for evaluating key wheat flour quality traits [32].

Building on the success of the GPT method in distinguishing wheat flour quality, and driven by the goal of streamlining the process by using wholemeal wheat directly, the fourth phase strategically shifted focus to evaluating wholemeal samples using the optimized protocol—eliminating the need for the labor-intensive milling step. Both 2021 and 2022 wheat registration wholemeal sample sets exhibited similar biplot patterns, with some variation likely attributable to genotype × environment interactions. A significant reduction in PMT was observed, which could be explained by fiber influence, enhancing gluten aggregation kinetics, as suggested by Goldestein et al. [28]. Notably, the distinct differences in BEM values between the HRS and HRW wheat classes—previously observed in refined flour samples—remained consistent in the wholemeal samples. The GPT parameters demonstrated distinct predictive capabilities across wheat classes, reinforcing the decision to evaluate HRS and HRW wheat separately. Several quality traits, including Farinograph parameters and bread baking performance, were reliably predicted by the wholemeal GPT data. The quality characteristics of wheat breeding lines, measured by Mixograph or Glutomatic, were also effectively predicted by wholemeal GPT data. Consistent with our findings, previous studies have also reported strong predictive performance of GPT with Farinograph STAB and ABS [12,33,34] when testing wholemeal wheat. Evaluating durum breeding material, a study [16] showed that GPT PMT well predicted gluten strength of tested lines and significantly correlated with Mixograph and Glutomatic data using wholemeal samples. Overall, our GPT wholemeal protocol predicted the HRS wheat quality traits more stronger than HRW wheat.

The final stage of GPT protocol development involved testing a stronger ionic solvent, 0.5 M CaCl_2_. It has been demonstrated that kosmotropic cations such as K^+^ and Na^+^, and chaotropic cations such as Mg^2+^ and Ca^2+^ influence gluten protein composition and functional properties [35]. As anticipated, PMT decreased, aligning with previous findings [8,36], testing various salt concentrations. They revealed that PMT tends to remain stable at low concentrations of the Hofmeister series but differences significantly at higher concentrations, with Ca^2+^ producing the lowest PMT, probably due to enhanced glutenin solubility and accelerated gluten network formation. Notably, in our study, the use of CaCl_2_ improved repeatability and strengthened correlations between GPT and quality parameters. For example, the correlation between Mixograph peak time and GPT PMT increased from r = 0.660 in the NaCl system to r = 0.874 in the CaCl_2_ system. Additionally, more significant correlations were observed between quality traits and GPT parameters.

In our study, the calculation of the GSI, as proposed by Wang et al. [25] to address variability in flour samples with differing Farinograph ABS values, showed stronger correlations than BEM for the flour protocol. However, the GPT wholemeal protocol did not yield significant improvements. For instance, in the 2024 HRW wheat registration sample set—where Farinograph ABS ranged from 54.8% to 60.9%—most associations between the GSI (calculated as BEM × A34) and other quality traits were weaker than those observed using BEM. In contrast, a newly introduced parameter in our study, calculated as PM-AM, demonstrated strong associations with several key quality traits, including Farinograph ABS, Mixograph peak value, Glutomatic dry gluten content, and bread volume. This suggests that PM-AM may serve as a promising new GPT-derived parameter for evaluating gluten quality, particularly when using wholemeal wheat.

Overall, by evaluating a broad range of genotypes across seven harvest years and diverse environments, we gained deeper insights into the relationships between GPT parameters and key quality traits. This has enabled more informed strategies for integrating GPT into the quality screening of wheat breeding lines. However, GPT’s capabilities are limited to assessing gluten strength and do not extend to analyzing gluten composition or profile. It is well established that the level of glutenin, especially insoluble fraction, and its ratio to gliadin correlate with dough strength and are indicative of superior bread making quality [20,21,23,29]. To address this limitation, we adopted an innovative approach by integrating a rapid protein fractionation (PF) method with GPT. The observed variations for gluten composition among various tested sample sets could be partly due to different protein content. However, the contribution of sub-protein fractions to the increase/decrease in protein content is not equal. It is reported that the insoluble glutenin level is not significantly affected by protein content [37,38]. Our results showed strong associations, for both wheat classes, between insoluble glutenin% and the ratio of insoluble to soluble glutenin with other quality traits, including GPT data, validating the integration of the PF protocol into the GPT wholemeal protocol. Our findings are consistent with a report [5] that examined the gluten strength using a GPT technique and through fractionation/reconstitution of flour/gluten. They found BEM to be more important in differentiating the gluten strength, while PMT was the primary parameter in differentiating the glutenin to gliadin ratios. Marti et al. [39] also reported significant correlations between GPT parameters and gluten fractions, measured by a labor-intensive sequential fractionation protocol and HPLC analysis. They found a trend in longer PMT and greater total area for the group of winter wheat flour samples with lower gliadin%, and higher glutenin and glutenin macropolymer (GMP) content. In our study, for both HRS and HRW wheat, a strong correlation was observed between GPT AM and insoluble glutenin%, consistent with the suggestion that AM might reflect the content of glutenin in wheat samples [7]. Overall, the associations between the PF data and quality traits were stronger for HRW wheat compared to HRS wheat. Since the GPT wholemeal protocol is better suited for the HRS wheat class, dual testing with PF allows for a more comprehensive assessment of physiochemical properties of gluten in diverse wheat types.

The GPT technique is grounded in the physical assessment of gluten aggregation behavior during high shear water–flour mixing. When combined with the PF method, it enhances the ability to screen for superior wheat quality lines. Supporting this integrated approach, one study reported a significantly higher prediction accuracy for bread baking quality (r = 0.70) when GPT data were combined with sedimentation values and protein content [14]. Another study [15] highlighted that pairing GPT with microscale extension testing yielded the most robust predictions of baking quality for vital gluten by-products, particularly in terms of BEM and PMT values.

As a representative example of practical applications of the developed protocols in a breeding context, we highlighted sample 38 (Figure 5a), grown across eight locations in the 2021 HRS wheat trials in Quebec. This line exhibited a higher Farinograph ABS (66% vs. 65%) and slightly lower bread volume (786 cm^3^ vs. 842 cm^3^) compared to the average values of strong check cultivars, Touran and Helios, in those trials. The line was supported for registration by QRCC based on its high flour protein content, strong Farinograph ABS, low MTI, and acceptable bread-making quality. The GPT wholemeal protocol for the HRS class predicted these outcomes by showing a positive correlation between BEM and bread volume, and a negative correlation between PMT and Farinograph ABS. The line had a lower PMT (51 s vs. 56 s) and lower BEM (70 BU vs. 78 BU) than the checks’ average. This line was later registered under the variety name “Arkco” (Registered Variety: Arkco—Canadian Food Inspection Agency). Notably, its performance in the 2023 Quebec wheat registration trials was as strong as checks. As a result, it was selected as a new check cultivar for Quebec HRS trials, replacing Touran in 2024—supporting the predictive utility of the GPT protocol in breeding selection. In contrast, sample 32 from the same set, despite having a similar flour protein content to the checks, did not meet the required functional quality standards and was not supported for registration. The GPT results for this sample also reflected weaker gluten characteristics, with longer PMT, lower BEM, and lower A34 values than the checks—demonstrating alignment between GPT-based predictions and final selection outcomes.

Sample 3 in the 2024 HRW registration trials in Ontario, is another example of validating the effectiveness of the screening protocols for identifying high-performing wheat lines. This sample demonstrated balanced and strong quality traits compared to the average of two check varieties, Pro 81 and Adrianus. Notably, it showed improved Farinograph ABS (57.6% vs. 56.2%) and greater bread volume (724 cm^3^ vs. 637 cm^3^) than the checks’ average, but lower Farinograph STAB (7.1 min vs. 14.0 min). The line received registration support from OCCC in 2024. The GPT and PF results confirmed these advantages (Figure 9). This line had a higher GPT PM-AM compared to the checks’ average (21 BU vs. 17 BU) which linked to higher Farinograph ABS; slightly lower BEM (63 BU vs. 69 BU) and A34 (632 vs. 706), which linked to lower Farinograph STAB; and comparable levels of insoluble glutenin and its ratio to soluble glutenin (42% and 0.75, respectively), which could explain its great bread baking performance.

## 5. Conclusions

Using wholemeal wheat, two high-throughput techniques, GlutoPeak (GPT) and protein fractionation (PF), were integrated to evaluate wheat gluten strength. Our study included a broad range of genotypes tested over seven harvest years and diverse environments, strengthening the understanding of how GPT parameters relate to other key quality traits. This extensive dataset enhances the reliability of GPT as a screening tool. Our protocols demonstrated strong potential for ranking breeding materials, with performance comparable to Farinograph, Mixograph, and Glutomatic. The optimized methods effectively differentiate wheat populations based on gluten strength and could assess the impact of biotic and abiotic stresses on flour protein functionality.

By eliminating the milling step, our approach indirectly reduces analysis cost. Additionally, the smaller sample size requirements further contribute to cost saving in breeding programs. The GPT protocol uses only 8 g of wholemeal wheat and allows up to ten runs per hour—significantly higher throughput than Farinograph and Mixograph, which require 50 g and 35 g of refined flour and accommodate approximately two and four runs per hour, respectively. Similarly, the optimized PF technique, requiring just 20 mg of wholemeal, enables up to 50 analyses per day for total or insoluble glutenin, further supporting its integration with the GPT protocol.

As with other empirical rheological tools, GPT parameters should be interpreted holistically to capture the full spectrum of gluten aggregation behavior. Applying GPT within a specific wheat class, alongside benchmark values from established checks, emerges as a highly effective strategy for ranking breeding lines based on gluten performance.

## Figures and Tables

**Table 1:** List of check wheat varieties included in the tested sample sets.

Year	Wheat Class	Region	Check Variety Name/s
2018	HRW HRW	Ontario Registration Trials Quebec Registration Trials	AC Morley and Gallus Lexington
	HRS HRS	Ontario Registration Trials Quebec Registration Trials	AC Carberry and Norwell Helios
2020	HRS	Advanced Breeding Trials	AAC Scotia, AC Helena, and AC Walton
2021	HRW HRW	Ontario Registration Trials Quebec Registration Trials	Pro 81 and Gallus Lexington
	HRS HRS HRS	Ontario Registration Trials Quebec Registration Trials Maritimes Registration Trials	AC Carberry and Ventry Helios and Touran AC Helena
2022	HRW HRW	Ontario Registration Trials Quebec Registration Trials	Pro 81 and Gallus Lexington
	HRW	Advanced Breeding Trials	Adrianus, Pro 81, and Gallus
	HRS HRS HRS	Ontario Registration Trials Quebec Registration Trials Maritimes Registration Trials	AC Carberry and Ventry Helios and Touran AC Helena and AC Walton
	HRS HRS	Advanced Breeding Trials Preliminary Breeding Trials	AAC Scotia, AC Helena, and Ventry AAC Scotia and Ventry
2023	HRW	Advanced Breeding Trials	PRO 81 and Adrianus
	HRS	Advanced Breeding Trials	AAC Scotia, AC Walton, and Ventry
2024	HRW HRW	Ontario Registration Trials Quebec Registration Trials	PRO 81 and Adrianus Lexington and Mirador
	HRW	Advanced Breeding Trials	PRO 81 and Adrianus
	HRS	Advanced Breeding Trials	AAC Scotia, Raven, and Ventry

**Table 2:** Details of the six methods tested during the initial stage of the GlutoPeak Tester (GPT) protocol development.

Method Number	Reference	Flour (g):Solvent (g)	Salt/Solution Type	Temperature (°C)	Speed (RPM)	Run Time (s)
1	Brabender method	8: 9	1.6% (*w*/*v*) NaCl	36	2750	300
2	Brabender method, adjusted for P% and M%	8: 9	1.6% (*w*/*v*) NaCl	36	2750	300
3	[7]	10.2: 11.2	1.0% (*w*/*v*) NaCl	36	2750	300
4	[24]	9: 10	2.0% (*w*/*v*) NaCl	35	3000	600
5	[14]	9: 10	Distilled H_2_O	35	2200	600
6	[25]	8: 10	Distilled H_2_O	34	2700	350

**Table 3:** Results of the six methods tested during the initial stage of GPT protocol development.

Sample @	GPT Parameters	Method#1 *	Method#2	Method#3	Method#4	Method#5	Method#6
1	PMT	119.7 ± 3.0	56.7 ± 2.2	100.0 ± 0.7	96.0 ± 0.0	176.0 ± 3.0	318.0 ± 9.0
2		103.0 ± 5.0	58.5 ± 1.5	82.3 ± 1.2	86.0 ± 1.0	99.0 ± 4.5	198.2 ± 12.0
3		126.3 ± 4.1	83.7 ± 4.4	100.3 ± 2.4	100.3 ± 0.9	169.3 ± 2.4	251.3 ± 1.2
4		295.0 ± 3.5	16.5 ± 0.5	173.7 ± 3.4	377.3 ± 26.7	145.5 ± 4.5	257.0 ± 11.2
1	BEM	53.0 ± 0.6	65.0 ± 0.6	71.3 ± 0.9	68.0 ± 0.0	51.7 ± 0.7	36.5 ± 0.5
2		55.0 ± 1.0	68.0 ± 2.0	78.0 ± 1.0	74.0 ± 0.6	60.2 ± 1.4	47.3 ± 0.9
3		51.3 ± 0.3	60.0 ± 0.9	72.0 ± 1.5	69.0 ± 0.6	52.3 ± 0.9	42.3 ± 0.3
4		19.7 ± 6.8	97.5 ± 0.5	38.0 ± 0.0	33.3 ± 0.3	28.5 ± 0.5	21.0 ± 1.0
1	AM	23.3 ± 0.9	43.0 ± 3.5	27.0 ± 0.6	24.5 ± 0.5	25.7 ± 2.6	16.0 ± 0.0
2		38.0 ± 1.0	41.0 ± 3.0	28.7 ± 0.3	28.0 ± 1.0	44.6 ± 3.6	18.2 ± 0.6
3		26.0 ± 2.0	30.7 ± 1.8	27.0 ± 1.5	26.7 ± 0.9	27.3 ± 0.9	17.3 ± 0.9
4		9.3 ± 2.9	N/R	20.0 ± 0.6	17.7 ± 0.3	11.5 ± 0.5	7.5 ± 0.5
1	PM	38.0 ± 0.6	51.7 ± 1.2	54.7 ± 0.7	50.0 ± 0.0	41.0 ± 0.0	29.0 ± 1.0
2		41.0 ± 0.0	52.0 ± 2.0	60.0 ± 0.6	56.0 ± 1.0	49.0 ± 3.9	35.8 ± 2.6
3		39.3 ± 0.9	46.7 ± 0.7	56.0 ± 0.6	52.0 ± 1.2	42.7 ± 0.9	32.3 ± 0.3
4		N/R	57.5 ± 8.5	31.3 ± 0.3	28.3 ± 0.3	22.5 ± 0.5	15.5 ± 0.5
1	A34	652 ± 13	901 ± 9	784 ± 15	672 ± 14	633 ± 55	390 ± 5
2		771 ± 9	913 ± 42	854 ± 22	834 ± 40	871 ± 46	527 ± 15
3		665 ± 17	786 ± 17	783 ± 40	803 ± 11	699 ± 10	478 ± 26
4		253 ± 84	948 ± 40	488 ± 8	424 ± 2	310 ± 3	230 ± 19
1	A45	695 ± 10	861 ± 30	952 ± 17	903 ± 6	704 ± 11	501 ± 13
2		715 ± 2	913 ± 1	1067 ± 10	1000 ± 23	811 ± 29	592 ± 25
3		695 ± 18	799 ± 6	988 ± 8	922 ± 19	724 ± 10	538 ± 5
4		975 ± 136	1257 ± 28	525 ± 2	471 ± 3	385 ± 3	287 ± 12
1	GSI ^	101,932 ± 3806	125,276 ± 1384	171,556 ± 4257	136,952 ± 2312	158,709 ± 6441	100,836 ± 8424
2		116,364 ± 2748	129,877 ± 7183	173,438 ± 4564	158,637 ± 3506	161,820 ± 5132	105,139 ± 3541
3		100,131 ± 1853	119,429 ± 1831	172,115 ± 14,007	150,849 ± 7672	153,576 ± 5584	100,839 ± 4457
4		20,999 ± 8468	92,499 ± 4423	65,220 ± 2480	74,608 ± 4464	29,745 ± 1633	16,401 ± 2981

Data are the average of three repeats ± SE. N/R, not registered by the software. * Method details shown in Table 1. ^ GSI = BEM × total area (A01 + A12 + A23 + A34). @ Sample: (1) A commercial all-purpose flour (Robin Hood, Toronto, Canada) with 12.13% protein and 12.25% moisture. (2) An AACCI check flour sample, HW-4 with 12.38% protein and 10.65% moisture. (3) An AACCI check flour sample, MIXO-1 with 13.03% protein, and 12.55% moisture. (4) An AACCI check flour sample for SRC testing with 8.08% protein and 12.10% moisture.

**Table 4:** Comparison of 1.0% and 1.6% NaCl solvents in GPT protocol development using wheat flour samples.

Sample	GPT Parameter	1.0% NaCl	1.6% NaCl
AACCI Hard Wheat	PMT	115.0 ± 0.7	116.3 ± 1.7
	BEM	63.0 ± 0.4	62.3 ± 0.6
	A23	813 ± 77	809 ± 39
	A34	753 ± 10	801 ± 5
	A45	857 ± 8	873 ± 3
	GSI	98,757 ± 5742	100,173 ± 1655
Hard Red Spring Wheat	PMT	139.8 ± 1.1	148.3 ± 2.1
	BEM	59.3 ± 0.5	58.5 ± 0.3
	A23	1191 ± 63	1329 ± 54
	A34	796 ± 6	801 ± 8
	A45	806 ± 2	815 ± 10
	GSI	117,811 ± 4951	124,564 ± 2807
Hard Red Winter Wheat	PMT	123.8 ± 1.4	135.3 ± 0.8
	BEM	46.3 ± 0.3	46.3 ± 0.3
	A23	938 ± 58	991 ± 42
	A34	576 ± 9	579 ± 13
	A45	632 ± 9	649 ± 7
	GSI	69,966 ± 2201	72,626 ± 2613

Data are the average of four repeats ± SE. GSI = BEM × (A23 + A34). The hard red spring and hard red winter flour samples were from a bulk made from the 2020 wheat registration check varieties in Ontario and Quebec trials.

**Table 5:** Comparison of 1% NaCl and 0.5 M CaCl_2_ solutions as the solvent in GPT protocol development using wholemeal wheat.

Sample	GPT Parameter	1% NaCl	0.5 M CaCl_2_
Hard Red Spring Wheat	PMT	71.5 ± 2.3	43.3 ± 0.3
	BEM	63.5 ± 1.6	68.7 ± 0.9
	AM	24.2 ± 0.4	24.2 ± 0.5
	PM	43.3 ± 0.7	45.0 ± 0.6
	PM-AM	19.2 ± 0.5	20.8 ± 0.6
	A34	630 ± 10	686 ± 9
	GSI	40,094 ± 1684	47,105 ± 1111
Hard Red Winter Wheat	PMT	68.7 ± 3.6	42.8 ± 0.5
	BEM	54.5 ± 1.4	57.2 ± 0.9
	AM	21.3 ± 0.9	19.8 ± 0.5
	PM	35.7 ± 0.6	37.0 ± 0.4
	PM-AM	14.3 ± 0.6	17.2 ± 0.7
	A34	562 ± 15	560 ± 10
	GSI	30,718 ± 1610	31,975 ± 638

Data are the average of six repeats ± SE. GSI = BEM × A34. The hard red spring and hard red winter samples were from a bulk made from the 2023 wheat registration check varieties in Ontario and Quebec trials.

## Data Availability

All data reported in this paper will be shared by the lead contact upon request.

## Supplementary Materials

The following supporting information can be downloaded at https://www.mdpi.com/article/10.3390/mps8050124/s1. Table S1: Correlation coefficients (r) among tested parameters for 2018 HRW wheat flour samples (n = 24). Values > 0.413 or <−0.413 are statistically significant (*p* < 0.05). Table S2: Correlation coefficients (r) among tested parameters for 2018 HRS wheat registration flour samples (n = 43). Values > 0.284 or <−0.284 are statistically significant (*p* < 0.05). Table S3: Correlation coefficients (r) among tested parameters for 2020 HRS wheat flour samples from Advanced breeding lines (n = 40). Values > 0.318 or <−0.318 are statistically significant (*p* < 0.05). Table S4: Correlation coefficients (r) among tested parameters for 2021 HRS wheat registration samples (n = 48). Values > 0.292 or <−0.292 are statistically significant (*p* < 0.05). Table S5: Correlation coefficients (r) among tested parameters for 2021 HRW wheat registration samples (n = 23). Values > 0.390 or <−0.390 are statistically significant (*p* < 0.05). Table S6: Correlation coefficients (r) among tested parameters for 2022 HRS wheat registration samples (n = 39). Values > 0.298 or <−0.298 are statistically significant (*p* < 0.05). Table S7: Correlation coefficients (r) among tested parameters for 2022 HRW wheat registration samples (n = 26). Values > 0.396 or <−0.396 are statistically significant (*p* < 0.05). Table S8: Correlation coefficients (r) among tested parameters for 2022 HRS Advanced wheat breeding lines (n = 19). Values > 0.430 or <−0.430 are statistically significant (*p* < 0.05). Table S9: Correlation coefficients (r) among tested parameters for 2022 HRS Preliminary wheat breeding lines (n = 64). Values > 0.251 or <−0.251 are statistically significant (*p* < 0.05). Table S10: Correlation coefficients (r) among tested parameters for 2023 HRW Advanced wheat breeding lines (n = 18). Values > 0.478 or <−0.478 are statistically significant (*p* < 0.05). Table S11: Correlation coefficients (r) among tested parameters for 2024 HRW wheat registration samples from Ontario (n = 11) and Quebec (n = 12) trials. Values > 0.390 or <−0.390 are statistically significant (*p* < 0.05). Table S12: Correlation coefficients (r) among tested parameters for 2024 HRW Advanced wheat breeding lines (n = 14). Values > 0.539 or <−0.539 are statistically significant (*p* < 0.05). Table S13: Correlation coefficients (r) among tested parameters for 2024 HRS Advanced wheat breeding lines (n = 24). Values > 0.413 or <−0.413 are statistically significant (*p* < 0.05). Table S14: Correlation coefficients (r) among tested parameters for 2021 HRW Advanced wheat breeding lines (n = 17). Values > 0.506 or <−0.506 are statistically significant (*p* < 0.05). Table S15: Correlation coefficients (r) among tested parameters for 2023 HRS Advanced wheat breeding lines (n = 42). Values > 0.311 or <−0.311 are statistically significant (*p* < 0.05).

## Abbreviations

The following abbreviations are used in this manuscript:

AAFC	Agriculture and Agri-Food Canada
ARCCC	Atlantic Recommending Committee for Cereal Crop
ABS	Farinograph Absorption
MTI	Farinograph Mixing Tolerance Index
PT	Farinograph Peak Time
STAB	Farinograph Stability
GPT	GlutoPeak Tester
PM-AM	GPT, Differences Between PM and AM Values
GSI	GPT, Gluten Strength Index
HRS	Hard Red Spring
HRW	Hard Red Winter
OCCC	Ontario Cereal Crop Committee
ORDC	Ottawa Research and Development Centre
PF	Protein Fractionation
QRCC	Quebec Recommending Committee for Cereals
