# Supplementary material for: Accelerated Screening of Wheat Gluten Strength Using Dual Physicochemical Tests in Diverse Breeding Lines
Source: Methods Protoc. 2025 Oct 18;8(5):124. doi: 10.3390/mps8050124 (PMC12566020; doi:10.3390/mps8050124)
Supplement: Supplementary file 1 [file mps-08-00124-s001.zip › mps-3820076-supplementary.pdf]

Supplemental Tables

Table S1. Correlation coefficients (r) among tested parameters for 2018 HRW wheat registration flour samples (n = 24). Values > 0.413 or < -0.413 are statistically significant (p < 0.05).

Entries	ABS	BVOL	FP	M-A03	M-A34	M-A35	M-A45	M-AM	M-BEM	M-GSI	M-PM	M-PMT	M-TArea	MTI	O-A03	O-A34	O-A35	O-A45	O-AM	O-BEM	O-GSI	O-PM	O-PMT	O-TArea	PT
**BVOL**	0.169	1																							
**FP**	0.346	0.650	1																						
**M-A03**	-0.265	0.365	-0.043	1																					
**M-A34**	0.656	0.639	0.594	0.171	1																				
**M-A35**	0.796	0.590	0.629	-0.001	0.950	1																			
**M-A45**	0.858	0.467	0.594	-0.192	0.783	0.938	1																		
**M-AM**	0.183	0.549	0.282	0.595	0.743	0.548	0.266	1																	
**M-BEM**	0.850	0.314	0.597	-0.452	0.645	0.811	0.900	0.122	1																
**M-GSI**	0.067	0.576	0.230	0.915	0.519	0.386	0.194	0.765	-0.066	1															
**M-PM**	0.796	0.484	0.489	-0.023	0.784	0.916	0.955	0.370	0.807	0.344	1														
**M-PMT**	-0.468	0.311	-0.078	0.940	-0.025	-0.189	-0.350	0.398	-0.598	0.778	-0.212	1													
**M-TArea**	-0.193	0.421	0.018	0.995	0.268	0.094	-0.109	0.657	-0.377	0.947	0.057	0.916	1												
**MTI**	-0.175	-0.613	-0.397	-0.385	-0.502	-0.454	-0.348	-0.491	-0.226	-0.550	-0.466	-0.347	-0.427	1											
**O-A03**	-0.087	0.327	0.157	0.862	0.261	0.168	0.044	0.530	-0.262	0.852	0.201	0.755	0.870	-0.334	1										
**O-A34**	0.325	0.304	0.227	0.249	0.446	0.431	0.364	0.423	0.240	0.394	0.320	0.100	0.289	-0.123	0.257	1									
**O-A35**	0.303	0.355	0.227	0.254	0.522	0.465	0.347	0.496	0.226	0.396	0.296	0.135	0.301	-0.175	0.168	0.927	1								
**O-A45**	0.179	0.327	0.159	0.185	0.485	0.379	0.217	0.462	0.138	0.282	0.170	0.150	0.230	-0.203	-0.019	0.537	0.815	1							
**O-AM**	0.262	0.282	0.051	0.580	0.498	0.423	0.289	0.598	0.049	0.690	0.405	0.365	0.618	-0.279	0.615	0.723	0.679	0.409	1						
**O-BEM**	0.313	0.363	0.322	-0.030	0.411	0.404	0.348	0.281	0.380	0.132	0.274	-0.091	0.012	-0.154	-0.152	0.606	0.780	0.815	0.391	1					
**O-GSI**	0.104	0.490	0.274	0.786	0.466	0.377	0.234	0.657	-0.044	0.865	0.338	0.634	0.815	-0.376	0.844	0.681	0.637	0.378	0.841	0.356	1				
**O-PM**	0.095	0.194	-0.102	0.477	0.339	0.205	0.031	0.519	-0.138	0.479	0.039	0.436	0.501	-0.275	0.263	0.556	0.720	0.760	0.534	0.466	0.519	1			
**O-PMT**	-0.134	0.527	0.434	0.729	0.293	0.217	0.107	0.464	-0.166	0.752	0.186	0.722	0.742	-0.412	0.866	0.122	0.056	-0.062	0.283	-0.174	0.685	0.129	1		
**O-TArea**	0.015	0.377	0.204	0.837	0.359	0.271	0.142	0.591	-0.164	0.869	0.269	0.699	0.855	-0.332	0.962	0.512	0.413	0.136	0.752	0.038	0.943	0.391	0.804	1	
**PT**	0.148	0.190	0.402	-0.399	0.268	0.375	0.451	-0.027	0.546	-0.173	0.405	-0.427	-0.363	-0.116	-0.249	0.167	0.105	-0.021	0.004	0.235	-0.089	-0.329	-0.152	-0.174	1
**STAB**	0.128	0.607	0.484	0.089	0.525	0.529	0.471	0.383	0.387	0.326	0.522	0.056	0.140	-0.749	0.117	0.240	0.247	0.183	0.278	0.288	0.270	0.024	0.236	0.173	0.644

Abbreviations: GPT parameters (PMT, BEM, AM, PM, A03, A34, A35, A45, TArea, GSI) are from the Ottawa location (O-) and Moulins lab (M-); flour protein (FP); Farinograph parameters include absorption (ABS), peak time (PT), stability (STAB), and mixing tolerance index (MTI); bread volume (BVOL). GSI = BEM x TArea.

Supplemental Tables

Table S2. Correlation coefficients (r) among tested parameters for 2018 HRS wheat registration flour samples (n = 43). Values > 0.284 or < -0.284 are statistically significant ($p < 0.05$).

Entries	ABS	BVOL	FP	M-A03	M-A34	M-A35	M-A45	M-AM	M-BEM	M-GSI	M-PM	M-PMT	M-TArea	MTI	O-A03	O-A34	O-A35	O-A45	O-AM	O-BEM	O-GSI	O-PM	O-PMT	O-TArea	PT
**BVOL**	0.216	1																							
**FP**	0.420	0.818	1																						
**M-A03**	-0.419	-0.019	-0.014	1																					
**M-A34**	0.814	0.273	0.360	-0.284	1																				
**M-A35**	-0.124	0.202	0.290	0.141	-0.446	1																			
**M-A45**	-0.279	0.123	0.184	0.185	-0.604	0.983	1																		
**M-AM**	0.535	0.423	0.491	0.104	0.795	-0.352	-0.478	1																	
**M-BEM**	0.855	0.198	0.280	-0.458	0.921	-0.444	-0.586	0.553	1																
**M-GSI**	0.074	0.135	0.176	0.812	0.309	-0.178	-0.223	0.538	0.137	1															
**M-PM**	0.593	0.497	0.590	-0.146	0.495	0.651	0.744	0.310	0.639	0.124	1														
**M-PMT**	-0.741	-0.164	-0.210	0.862	-0.696	0.256	0.371	-0.317	-0.784	0.434	-0.379	1													
**M-TArea**	-0.318	0.019	0.036	0.991	-0.153	0.084	0.106	0.217	-0.344	0.880	-0.097	0.792	1												
**MTI**	0.388	-0.037	0.035	-0.291	0.275	-0.211	-0.244	0.246	0.259	-0.122	0.099	-0.412	-0.261	1											
**O-A03**	-0.318	0.176	0.243	0.886	-0.328	0.393	0.418	0.070	-0.456	0.672	0.078	0.782	0.867	-0.294	1										
**O-A34**	0.412	-0.529	-0.497	-0.094	0.293	-0.172	-0.214	0.012	0.389	0.113	0.030	-0.270	-0.056	0.248	-0.169	1									
**O-A35**	0.421	-0.540	-0.526	-0.173	0.343	-0.226	-0.272	0.023	0.438	0.062	-0.005	-0.341	-0.130	0.250	-0.294	0.968	1								
**O-A45**	0.399	-0.512	-0.520	-0.249	0.374	-0.269	-0.317	0.034	0.460	-0.001	-0.044	-0.395	-0.205	0.234	-0.414	0.859	0.959	1							
**O-AM**	0.220	-0.130	-0.019	0.505	0.086	0.085	0.058	0.171	0.065	0.578	0.185	0.244	0.532	0.184	0.549	0.560	0.413	0.216	1						
**O-BEM**	0.370	-0.570	-0.599	-0.360	0.285	-0.237	-0.270	-0.125	0.421	-0.160	-0.085	-0.440	-0.332	0.236	-0.503	0.882	0.943	0.939	0.177	1					
**O-GSI**	-0.064	-0.198	-0.132	0.775	-0.091	0.189	0.187	0.097	-0.169	0.719	0.033	0.555	0.786	-0.139	0.777	0.458	0.355	0.213	0.779	0.125	1				
**O-PM**	0.377	-0.418	-0.374	-0.007	0.409	-0.265	-0.321	0.195	0.435	0.261	0.012	-0.241	0.050	0.244	-0.177	0.747	0.848	0.897	0.354	0.753	0.386	1			
**O-PMT**	-0.337	0.483	0.561	0.621	-0.411	0.576	0.598	-0.035	-0.510	0.346	0.220	0.642	0.583	-0.373	0.809	-0.598	-0.699	-0.760	0.171	-0.795	0.314	-0.593	1		
**O-TArea**	-0.260	0.080	0.154	0.889	-0.288	0.374	0.392	0.065	-0.404	0.702	0.084	0.753	0.876	-0.279	0.981	0.004	-0.126	-0.264	0.651	-0.354	0.874	-0.038	0.716	1	
**PT**	0.370	0.596	0.577	-0.206	0.348	0.031	-0.044	0.391	0.336	0.011	0.392	-0.302	-0.164	0.089	-0.039	-0.254	-0.222	-0.168	-0.147	-0.260	-0.218	-0.134	0.141	-0.099	1
**STAB**	-0.297	0.246	0.193	0.250	-0.161	0.185	0.198	-0.009	-0.187	0.148	0.039	0.351	0.235	-0.760	0.325	-0.402	-0.380	-0.326	-0.272	-0.385	0.066	-0.290	0.444	0.267	0.328

Abbreviations: GPT parameters (PMT, BEM, AM, PM, A03, A34, A35, A45, TArea, GSI) are from the Ottawa location (O-) and Moulins lab (M-); flour protein (FP); Farinograph parameters include absorption (ABS), peak time (PT), stability (STAB), and mixing tolerance index (MTI); bread volume (BVOL). GSI = BEM x TArea.

Table S3. Correlation coefficients (r) among tested parameters for 2020 HRS wheat flour samples from Advanced breeding lines (n = 40). Values > 0.318 or < -0.318 are statistically significant (p < 0.05).

Entries	A23	A34	AM	BEM	CTV	DryGlu	FP	GluIndex	GSI	Integ	PM	PMT	PT	PV	RPS	StrongGlu	WaterBind
**A34**	0.107	1															
**AM**	0.406	0.817	1														
**BEM**	-0.475	0.625	0.191	1													
**CTV**	0.531	0.046	0.179	-0.247	1												
**DryGlu**	-0.301	0.573	0.461	0.385	-0.293	1											
**FP**	-0.270	0.575	0.490	0.378	-0.321	0.930	1										
**GluIndex**	0.560	-0.094	0.103	-0.415	0.562	-0.573	-0.538	1									
**GSI**	0.918	0.463	0.616	-0.108	0.493	-0.110	-0.079	0.461	1								
**Integ**	-0.581	0.346	0.089	0.511	-0.195	0.330	0.261	-0.121	-0.379	1							
**PM**	-0.114	0.851	0.644	0.710	-0.128	0.646	0.661	-0.281	0.226	0.312	1						
**PMT**	0.904	-0.169	0.186	-0.668	0.619	-0.494	-0.472	0.671	0.716	-0.644	-0.396	1					
**PT**	0.842	-0.168	0.134	-0.53	0.668	-0.451	-0.392	0.546	0.680	-0.790	-0.279	0.900	1				
**PV**	-0.482	0.383	0.169	0.471	0.057	0.430	0.332	-0.142	-0.283	0.906	0.319	-0.523	-0.65	1			
**RPS**	-0.420	0.341	0.241	0.428	-0.48	0.582	0.541	-0.476	-0.154	0.626	0.391	-0.619	-0.771	0.649	1		
**StrongGlu**	0.128	0.590	0.624	0.100	0.128	0.678	0.630	0.211	0.269	0.289	0.518	-0.005	-0.067	0.383	0.255	1	
**WarterBind**	-0.553	0.354	0.166	0.428	-0.511	0.892	0.873	-0.747	-0.399	0.404	0.490	-0.713	-0.646	0.434	0.690	0.400	1
**WetGlu**	-0.487	0.431	0.263	0.425	-0.455	0.947	0.912	-0.710	-0.317	0.390	0.551	-0.661	-0.600	0.443	0.671	0.497	0.990

Abbreviations: GPT parameters include PMT, BEM, A23, A34, AM, PM, and GSI; Mixograph parameters include peak time (PT), peak value (PV), right of peak slope (RPS), curve tail value (CTV), and integral at the end of run (Integ); Glutomatic parameters include wet gluten (WetGlu), gluten index (GluIndex), dry gluten (DryGlu), water binging capacity (WaterBind), and strong gluten index (StrongGlu); flour protein (FP). GSI = BEM x (A23 + A34).

Supplemental Tables

Table S4. Correlation coefficients (r) among tested parameters for 2021 HRS wheat registration samples (n = 48). Values > 0.292 or < -0.292 are statistically significant (p < 0.05).

Entries	A34	ABS	AM	BEM	BVOL	FP	GSI	MTI	PM	PMT	PT
**ABS**	0.494	1									
**AM**	0.735	0.054	1								
**BEM**	0.764	0.722	0.228	1							
**BVOL**	0.560	0.685	0.185	0.749	1						
**FP**	0.585	0.786	0.093	0.854	0.866	1					
**GSI**	0.961	0.621	0.557	0.907	0.664	0.723	1				
**MTI**	-0.362	0.135	-0.556	-0.046	-0.126	-0.007	-0.249	1			
**PM**	0.844	0.657	0.439	0.937	0.759	0.824	0.927	-0.172	1		
**PMT**	-0.053	-0.566	0.285	-0.318	-0.282	-0.395	-0.160	-0.477	-0.284	1	
**PT**	0.355	0.407	0.045	0.584	0.579	0.569	0.448	-0.110	0.560	-0.162	1
**STAB**	0.416	-0.206	0.538	0.210	0.133	0.058	0.343	-0.793	0.299	0.554	0.433

Abbreviations: GPT parameters include PMT, BEM, A34, AM, PM, and GSI; flour protein (FP); Farinograph parameters include absorption (ABS), peak time (PT), stability (STAB), and mixing tolerance index (MTI); bread volume (BVOL). GSI = BEM x A34.

Supplemental Tables

Table S5. Correlation coefficients (r) among tested parameters for 2021 HRW wheat registration samples (n = 23). Values > 0.390 or < -0.390 are statistically significant ($p < 0.05$).

Entries	A34	ABS	AM	BEM	BVOL	FP	GSI	MTI	PM	PMT	PT
**ABS**	0.081	1									
**AM**	0.887	-0.182	1								
**BEM**	0.533	0.653	0.301	1							
**BVOL**	0.641	0.042	0.662	0.293	1						
**FP**	0.696	0.300	0.584	0.489	0.650	1					
**GSI**	0.914	0.364	0.726	0.829	0.559	0.691	1				
**MTI**	-0.376	0.232	-0.474	-0.137	-0.681	-0.195	-0.319	1			
**PM**	0.707	0.440	0.586	0.881	0.565	0.558	0.889	-0.474	1		
**PMT**	0.311	-0.626	0.558	-0.292	0.486	0.183	0.065	-0.483	-0.001	1	
**PT**	0.276	0.300	0.069	0.381	0.134	0.277	0.387	0.025	0.293	-0.270	1
**STAB**	0.440	-0.199	0.560	0.223	0.718	0.328	0.400	-0.860	0.522	0.565	0.061

Abbreviations: GPT parameters include PMT, BEM, A34, AM, PM, and GSI; flour protein (FP); Farinograph parameters include absorption (ABS), peak time (PT), stability (STAB), and mixing tolerance index (MTI); bread volume (BVOL). GSI = BEM x A34.

Table S6. Correlation coefficients (r) among tested parameters for 2022 HRS wheat registration samples (n = 39). Values > 0.298 or < -0.298 are statistically significant ($p < 0.05$).

Entries	A34	ABS	AM	BEM	BVOL	FP	GSI	MTI	PM	PMT	PT
**ABS**	0.576	1									
**AM**	0.459	0.133	1								
**BEM**	0.821	0.604	-0.042	1							
**BVOL**	0.683	0.560	0.187	0.631	1						
**FP**	0.695	0.712	-0.008	0.809	0.791	1					
**GSI**	0.940	0.613	0.173	0.963	0.683	0.787	1				
**MTI**	-0.349	0.141	-0.397	-0.076	-0.387	-0.088	-0.216	1			
**PM**	0.853	0.780	0.266	0.866	0.719	0.821	0.896	-0.159	1		
**PMT**	-0.341	-0.725	-0.013	-0.503	-0.269	-0.543	-0.436	-0.403	-0.620	1	
**PT**	0.410	0.379	0.009	0.479	0.471	0.603	0.451	-0.235	0.529	-0.249	1
**STAB**	0.352	-0.075	0.277	0.180	0.356	0.223	0.260	-0.765	0.231	0.333	0.620

Abbreviations: GPT parameters include PMT, BEM, A34, AM, PM, and GSI; flour protein (FP); Farinograph parameters include absorption (ABS), peak time (PT), stability (STAB), and mixing tolerance index (MTI); bread volume (BVOL). GSI = BEM x A34.

Table S7. Correlation coefficients (r) among tested parameters for 2022 HRW wheat registration samples (n = 26). Values > 0.396 or < -0.396 are statistically significant ($p < 0.05$).

Entries	A34	ABS	AM	BEM	BVOL	FP	GSI	MTI	PM	PMT	PT
**ABS**	0.391	1									
**AM**	0.739	0.332	1								
**BEM**	0.750	0.327	0.225	1							
**BVOL**	0.079	0.170	-0.061	0.096	1						
**FP**	0.146	0.597	-0.178	0.390	0.629	1					
**GSI**	0.927	0.390	0.506	0.939	0.117	0.297	1				
**MTI**	-0.011	0.211	-0.002	0.059	-0.639	-0.294	0.016	1			
**PM**	0.728	0.544	0.368	0.799	0.372	0.588	0.834	-0.258	1		
**PMT**	0.244	-0.614	0.051	0.277	0.263	-0.102	0.275	-0.507	0.151	1	
**PT**	0.200	0.413	0.010	0.227	0.586	0.663	0.228	-0.325	0.429	-0.092	1
**STAB**	-0.099	-0.135	-0.183	0.010	0.769	0.425	-0.028	-0.909	0.296	0.412	0.457

Abbreviations: GPT parameters include PMT, BEM, A34, AM, PM, and GSI; flour protein (FP); Farinograph parameters include absorption (ABS), peak time (PT), stability (STAB), and mixing tolerance index (MTI); bread volume (BVOL). GSI = BEM x A34.

Table S8. Correlation coefficients (r) among tested parameters for 2022 HRS Advanced wheat breeding lines (n = 19). Values > 0.430 or < -0.430 are statistically significant ($p < 0.05$).

Entries	A34	A45	AM	BEM	CTV	FP	GSI	Integ	PM	PMT	PT	PV
**A45**	0.272	1										
**AM**	0.772	-0.151	1									
**BEM**	0.482	0.903	-0.073	1								
**CTV**	0.096	0.457	-0.084	0.456	1							
**FP**	0.433	0.733	0.024	0.760	0.138	1						
**GSI**	0.887	0.647	0.450	0.832	0.303	0.672	1					
**Integ**	0.281	0.546	-0.118	0.696	0.799	0.387	0.544	1				
**PM**	0.534	0.917	0.073	0.943	0.478	0.775	0.836	0.637	1			
**PMT**	-0.548	-0.054	-0.337	-0.317	0.113	-0.352	-0.513	-0.293	-0.350	1		
**PT**	-0.283	-0.182	0.079	-0.432	-0.081	-0.315	-0.408	-0.624	-0.311	0.660	1	
**PV**	0.246	0.603	-0.177	0.726	0.809	0.438	0.539	0.989	0.665	-0.214	-0.574	1
**RPS**	0.061	0.526	-0.283	0.618	0.404	0.526	0.365	0.743	0.581	-0.395	-0.655	0.764

Abbreviations: GPT parameters include PMT, BEM, A34, A45, AM, PM, and GSI; flour protein (FP); Mixograph parameters include peak time (PT), peak value (PV), right of peak slope (RPS), curve tail value (CTV), and integral at the end of the run (Integ). GSI = BEM x A34.

Supplemental Tables

Table S9. Correlation coefficients (r) among tested parameters for 2022 HRS Preliminary wheat breeding lines (n = 64). Values > 0.251 or < -0.251 are statistically significant ($p < 0.05$).

Entries	A34	A45	AM	BEM	DryGlu	GluIndex	GSI	PM	PMT	StrongGlu	WaterBind
**A45**	0.662	1									
**AM**	0.561	0.159	1								
**BEM**	0.685	0.942	0.181	1							
**DryGlu**	0.224	0.570	-0.241	0.645	1						
**GluIndex**	0.323	0.124	0.353	-0.013	-0.359	1					
**GSI**	0.920	0.872	0.404	0.912	0.484	0.176	1				
**PM**	0.743	0.944	0.218	0.949	0.581	0.080	0.923	1			
**PMT**	0.041	0.010	0.205	-0.226	-0.491	0.668	-0.097	-0.135	1		
**StrongGlu**	0.477	0.590	0.106	0.529	0.515	0.611	0.563	0.558	0.192	1	
**WaterBind**	0.064	0.390	-0.319	0.505	0.945	-0.543	0.319	0.417	-0.646	0.303	1
**WetGlu**	0.116	0.452	-0.298	0.556	0.974	-0.491	0.375	0.474	-0.604	0.374	0.995

Abbreviations: GPT parameters include PMT, BEM, A34, A45, AM, PM, and GSI; Glutomatic parameters include wet gluten (WetGlu), gluten index (GluIndex), dry gluten (DryGlu), water binging capacity (WaterBind), and strong gluten index (StrongGlu). GSI = BEM x A34.

Supplemental Tables

Table S10. Correlation coefficients (r) among tested parameters for 2023 HRW Advanced wheat breeding lines (n = 18). Values > 0.478 or < -0.478 are statistically significant (p < 0.05).

Entries	A34	A45	AM	BEM	CTV	FP	GSI	IG%	IG/SG	Integ	PM	PMT	PT	PV	RPS
**A45**	0.793	1													
**AM**	0.739	0.498	1												
**BEM**	0.705	0.892	0.206	1											
**CTV**	0.609	0.667	0.384	0.642	1										
**FP**	0.086	0.201	-0.047	0.439	0.276	1									
**GSI**	0.907	0.908	0.473	0.937	0.665	0.305	1								
**IG%**	0.143	0.335	0.185	0.114	0.116	-0.168	0.119	1							
**IG/SG**	0.127	0.324	0.190	0.102	0.094	-0.158	0.105	0.994	1						
**Integ**	0.168	0.196	-0.189	0.339	0.440	0.130	0.276	0.173	0.153	1					
**PM**	0.878	0.946	0.510	0.935	0.684	0.297	0.980	0.167	0.148	0.221	1				
**PMT**	0.207	0.328	0.593	-0.097	0.142	-0.439	0.029	0.441	0.456	-0.347	0.117	1			
**PT**	0.194	0.316	0.487	0.021	0.186	-0.120	0.097	0.360	0.362	-0.469	0.195	0.819	1		
**PV**	0.094	0.176	-0.184	0.280	0.417	0.095	0.203	0.250	0.240	0.971	0.163	-0.209	-0.338	1	
**RPS**	0.034	-0.252	0.243	-0.304	-0.139	-0.157	-0.157	-0.372	-0.362	0.116	-0.183	-0.112	-0.456	0.048	1
**SG%**	-0.143	-0.335	-0.185	-0.114	-0.116	0.168	-0.119	-1	-0.994	-0.173	-0.167	-0.441	-0.360	-0.250	0.372

Abbreviations: GPT parameters include PMT, BEM, A34, A45, AM, PM, and GSI; Mixograph parameters include peak time (PT), peak value (PV), right of peak slope (RPS), curve tail value (CTV), and integral at the end of run (Integ); flour protein (FP); soluble glutenin% (SG%), insoluble glutenin% (IG%), insoluble/soluble ratio (IG/SG). GSI = BEM x A34.

Supplemental Tables

Table S11. Correlation coefficients (r) among tested parameters for 2024 HRW wheat registration samples from Ontario (n = 11) and Quebec (n = 12) trials. Values > 0.390 or < -0.390 are statistically significant ($p < 0.05$).

Entries	A34	A45	ABS	AM	BEM	BVOL	FP	GSI	IG%	IG/MP	IG/SG	MP%	MTI	PM	PMT	PT	SG%	STAB	TG%
**A45**	0.868	1																	
**ABS**	0.231	0.420	1																
**AM**	0.425	0.100	-0.463	1															
**BEM**	0.829	0.950	0.400	-0.036	1														
**BVOL**	0.172	0.137	0.576	-0.177	0.177	1													
**FP**	0.488	0.606	0.637	-0.201	0.622	0.590	1												
**GSI**	0.947	0.95	0.353	0.162	0.962	0.188	0.596	1											
**IG%**	0.232	0.044	-0.218	0.613	-0.090	0.189	0.104	0.057	1										
**IG/MP**	0.271	0	-0.385	0.814	-0.125	-0.037	-0.238	0.040	0.594	1									
**IG/SG**	0.255	0.064	-0.214	0.623	-0.071	0.178	0.114	0.081	0.998	0.594	1								
**MP%**	-0.096	0.07	0.266	-0.464	0.108	0.159	0.395	0.038	0.137	-0.702	0.136	1							
**MTI**	-0.501	-0.541	-0.327	0.127	-0.670	-0.509	-0.712	-0.631	0	0.060	-0.020	-0.073	1						
**PM**	0.815	0.947	0.563	0.021	0.864	0.186	0.601	0.877	-0.025	-0.098	-0.008	0.117	-0.414	1					
**PMT**	-0.067	-0.334	-0.500	0.823	-0.490	-0.225	-0.458	-0.330	0.589	0.732	0.591	-0.420	0.430	-0.362	1				
**PT**	0.594	0.589	0.475	-0.189	0.649	0.371	0.661	0.680	-0.018	-0.286	-0.004	0.361	-0.626	0.616	-0.516	1			
**SG%**	-0.232	-0.044	0.218	-0.613	0.090	-0.189	-0.104	-0.057	-1	-0.594	-0.998	-0.137	0	0.025	-0.589	0.018	1		
**STAB**	0.657	0.622	0.159	0.223	0.654	0.317	0.660	0.696	0.299	0.230	0.329	-0.016	-0.878	0.483	-0.130	0.615	-0.299	1	
**TG%**	0.096	-0.07	-0.266	0.464	-0.108	-0.159	-0.395	-0.038	-0.137	0.702	-0.136	-1	0.073	-0.117	0.420	-0.361	0.137	0.016	1
**TG/MP**	0.096	-0.07	-0.263	0.459	-0.110	-0.161	-0.394	-0.039	-0.134	0.707	-0.133	-0.999	0.077	-0.117	0.419	-0.366	0.134	0.012	0.999

Abbreviations: GPT parameters include PMT, BEM, A34, A45, AM, PM, and GSI; flour protein (FP); Farinograph parameters include absorption (ABS), peak time (PT), stability (STAB), and mixing tolerance index (MTI); bread volume (BVOL); total glutenin% (TG%); insoluble glutenin% (IG%); soluble glutenin% (SG%); monomeric protein% (MP%); the ratio of insoluble to soluble glutenin (IG/SG); the ratio of insoluble glutenin to monomeric protein (IG/MP). GSI = BEM x A34.

Table S12. Correlation coefficients (r) among tested parameters for 2024 HRW Advanced wheat breeding lines (n = 14). Values > 0.539 or < -0.539 are statistically significant (p < 0.05).

Entries	A34	A45	AM	BEM	CTV	FP	GSI	IG%	IG/SG	Integ	PM	PMT	PT	PV	RPS
**A45**	0.637	1													
**AM**	0.597	-0.074	1												
**BEM**	0.608	0.914	-0.214	1											
**CTV**	0.594	0.563	0.395	0.362	1										
**FP**	0.465	0.876	-0.205	0.808	0.223	1									
**GSI**	0.852	0.883	0.128	0.933	0.496	0.740	1								
**IG%**	0.414	0.283	0.432	0.096	0.234	0.321	0.252	1							
**IG/SG**	0.443	0.320	0.416	0.153	0.225	0.360	0.305	0.996	1						
**Integ**	0.223	0.303	-0.187	0.435	0.373	0.039	0.381	-0.321	-0.290	1					
**PM**	0.730	0.915	0.063	0.846	0.528	0.754	0.881	0.340	0.382	0.357	1				
**PMT**	0.537	0.348	0.633	0.106	0.562	0.223	0.313	0.606	0.576	-0.251	0.232	1			
**PT**	0.437	0.330	0.576	0.091	0.455	0.212	0.260	0.554	0.541	-0.481	0.265	0.770	1		
**PV**	0.245	0.417	-0.296	0.571	0.287	0.185	0.492	-0.119	-0.083	0.832	0.400	-0.045	-0.280	1	
**RPS**	-0.330	-0.696	-0.068	-0.454	-0.442	-0.644	-0.432	-0.325	-0.325	0.169	-0.559	-0.395	-0.579	0.209	1
**SG%**	-0.414	-0.283	-0.432	-0.096	-0.234	-0.321	-0.252	-1	-0.996	0.321	-0.340	-0.606	-0.554	0.119	0.325

Abbreviations: GPT parameters include PMT, BEM, A34, A45, AM, PM, and GSI; flour protein (FP); Mixograph parameters include peak time (PT), peak value (PV), right of peak slope (RPS), curve tail value (CTV), and integral at the end of the run (Integ); soluble glutenin% (SG%), insoluble glutenin% (IG%), the ratio of insoluble to soluble glutenin (IG/SG). GSI = BEM x A34.

Supplemental Tables

Table S13. Correlation coefficients (r) among tested parameters for 2024 HRS Advanced wheat breeding lines (n = 24). Values > 0.413 or < -0.413 are statistically significant ($p < 0.05$).

Entries	A34	A45	AM	BEM	CTV	FP	GSI	IG%	IG/SG	Integ	PM	PMT	PT	PV	RPS
**A34**	1														
**A45**	0.862	1													
**AM**	0.798	0.493	1												
**BEM**	0.743	0.911	0.272	1											
**CTV**	0.672	0.555	0.525	0.585	1										
**FP**	0.456	0.496	0.14	0.547	0.261	1									
**GSI**	0.927	0.940	0.557	0.935	0.667	0.569	1								
**IG%**	0.561	0.420	0.765	0.189	0.332	0.185	0.384	1							
**IG/SG**	0.543	0.400	0.756	0.173	0.300	0.189	0.368	0.996	1						
**Integ**	0.462	0.565	0.039	0.639	0.639	0.407	0.582	-0.172	-0.196	1					
**PM**	0.819	0.936	0.455	0.826	0.574	0.459	0.872	0.307	0.286	0.612	1				
**PMT**	0.360	0.076	0.721	-0.039	0.519	-0.123	0.163	0.699	0.684	-0.235	0.028	1			
**PT**	0.197	-0.041	0.571	-0.161	0.246	-0.176	0.018	0.644	0.634	-0.548	-0.104	0.874	1		
**PV**	0.455	0.569	0.012	0.667	0.605	0.419	0.589	-0.185	-0.203	0.978	0.592	-0.264	-0.564	1	
**RPS**	-0.044	0.161	-0.457	0.303	-0.137	0.242	0.137	-0.521	-0.503	0.500	0.136	-0.730	-0.783	0.615	1
**SG%**	-0.561	-0.420	-0.765	-0.189	-0.332	-0.185	-0.384	-1	-0.996	0.172	-0.307	-0.699	-0.644	0.185	0.521

Abbreviations: GPT parameters include PMT, BEM, A34, A45, AM, PM, and GSI; flour protein (FP); Mixograph parameters include peak time (PT), peak value (PV), right of peak slope (RPS), curve tail value (CTV), and integral at the end of the run (Integ); soluble glutenin% (SG%), insoluble glutenin% (IG%), the ratio of insoluble to soluble glutenin (IG/SG). GSI = BEM x A34.

Supplemental Tables

Table S14. Correlation coefficients (r) among tested parameters for 2021 HRW Advanced wheat breeding lines (n = 17). Values > 0.506 or < -0.506 are statistically significant ($p < 0.05$).

Entries	CTV	FP	IG%	IG/SG	Integ	MP%	PT	PV	RPS	SG%	TG%
**FP**	0.735	1									
**IG%**	0.766	0.634	1								
**IG/SG**	0.813	0.673	0.964	1							
**Integ**	0.519	0.61	0.529	0.462	1						
**MP%**	-0.283	-0.422	-0.403	-0.419	-0.449	1					
**PT**	0.568	0.486	0.732	0.739	0.028	-0.260	1				
**PV**	0.848	0.819	0.752	0.767	0.750	-0.395	0.442	1			
**RPS**	-0.465	-0.338	-0.345	-0.380	-0.036	0.013	-0.277	-0.184	1		
**SG%**	-0.766	-0.634	-1	-0.964	-0.529	0.403	-0.732	-0.752	0.345	1	
**TG%**	0.283	0.422	0.403	0.419	0.449	-1	0.260	0.395	-0.013	-0.403	1
**TG/MP**	0.287	0.422	0.410	0.427	0.452	-0.998	0.265	0.400	-0.031	-0.410	0.998

Abbreviations: flour protein (FP); Mixograph parameters include peak time (PT), peak value (PV), right of peak slope (RPS), curve tail value (CTV), and integral at the end of the run (Integ); total glutenin% (TG%); soluble glutenin% (SG%), insoluble glutenin% (IG%), the ratio of insoluble to soluble glutenin (IG/SG); monomeric protein% (MP%); the ratio of insoluble glutenin to monomeric protein (IG/MP).

Table S15. Correlation coefficients (r) among tested parameters for 2023 HRS Advanced wheat breeding lines (n = 42). Values > 0.311 or < -0.311 are statistically significant ($p < 0.05$).

Entries	CTV	FP	IG%	IG/SG	Integ	PT	PV	RPS
**FP**	0.130	1						
**IG%**	0.452	-0.115	1					
**IG/SG**	0.486	-0.081	0.989	1				
**Integ**	0.769	0.159	0.229	0.256	1			
**PT**	0.354	0.126	0.345	0.363	-0.262	1		
**PV**	0.726	0.283	0.170	0.204	0.967	-0.276	1	
**RPS**	0.261	0.399	-0.167	-0.143	0.696	-0.549	0.819	1
**SG%**	-0.452	0.115	-1	-0.989	-0.229	-0.345	-0.170	0.167

Abbreviations: flour protein (FP); Mixograph parameters include peak time (PT), peak value (PV), right of peak slope (RPS), curve tail value (CTV), and integral at the end of the run (Integ); Soluble glutenin% (SG%), insoluble glutenin% (IG%), the ratio of insoluble to soluble glutenin (IG/SG). GSI = BEM x A34.
